# Multimorbidity and Patient Safety Incidents in Primary Care: A Systematic Review and Meta-Analysis

**DOI:** 10.1371/journal.pone.0135947

**Published:** 2015-08-28

**Authors:** Maria Panagioti, Jonathan Stokes, Aneez Esmail, Peter Coventry, Sudeh Cheraghi-Sohi, Rahul Alam, Peter Bower

**Affiliations:** 1 NIHR School for Primary Care Research, Centre for Primary Care, Manchester Academic Health Science Centre, University of Manchester, Manchester, United Kingdom; 2 NIHR Greater Manchester Primary Care Patient Safety Translational Research Centre (Greater Manchester PSTRC), Manchester Academic Health Science Centre University of Manchester, Manchester, United Kingdom; 3 NIHR Collaboration for Leadership in Applied Health Research and Care—Greater Manchester and Manchester Academic Health Science Centre, University of Manchester, Manchester, United Kingdom; Institute of Tropical Medicine (NEKKEN), Nagasaki University, JAPAN

## Abstract

**Background:**

Multimorbidity is increasingly prevalent and represents a major challenge in primary care. Patients with multimorbidity are potentially more likely to experience safety incidents due to the complexity of their needs and frequency of their interactions with health services. However, rigorous syntheses of the link between patient safety incidents and multimorbidity are not available. This review examined the relationship between multimorbidity and patient safety incidents in primary care.

**Methods:**

We followed our published protocol (PROSPERO registration number: CRD42014007434). Medline, Embase and CINAHL were searched up to May 2015. Study design and quality were assessed. Odds ratios (OR) and 95% confidence intervals (95% CIs) were calculated for the associations between multimorbidity and two categories of patient safety outcomes: ‘active patient safety incidents’ (such as adverse drug events and medical complications) and ‘precursors of safety incidents’ (such as prescription errors, medication non-adherence, poor quality of care and diagnostic errors). Meta-analyses using random effects models were undertaken.

**Results:**

Eighty six relevant comparisons from 75 studies were included in the analysis. Meta-analysis demonstrated that physical-mental multimorbidity was associated with an increased risk for ‘active patient safety incidents’ (OR = 2.39, 95% CI = 1.40 to 3.38) and ‘precursors of safety incidents’ (OR = 1.69, 95% CI = 1.36 to 2.03). Physical multimorbidity was associated with an increased risk for active safety incidents (OR = 1.63, 95% CI = 1.45 to 1.80) but was not associated with precursors of safety incidents (OR = 1.02, 95% CI = 0.90 to 1.13). Statistical heterogeneity was high and the methodological quality of the studies was generally low.

**Conclusions:**

The association between multimorbidity and patient safety is complex, and varies by type of multimorbidity and type of safety incident. Our analyses suggest that multimorbidity involving mental health may be a key driver of safety incidents, which has important implication for the design and targeting of interventions to improve safety. High quality studies examining the mechanisms of patient safety incidents in patients with multimorbidity are needed, with the goal of promoting effective service delivery and ameliorating threats to safety in this group of patients.

## Introduction

Primary care is increasingly responsible for the care of patients with long-term conditions, and improving the quality of their care is a major policy priority [1]. Patient safety is an essential component of high quality of care. Patient safety is defined as the ‘avoidance, prevention, and amelioration of adverse outcomes or injuries stemming from the processes of health care’[2]. Patient safety incidents are viewed as ‘any unintended events or hazardous conditions resulting from the process of care, rather than due to the patient's underlying disease, that led or could have led to unintended health consequences for the patient or health care processes linked to safety outcomes’[3]. Patient safety incidents can occur during access to care, clinical delivery (i.e. adverse drug events, prescription errors, diagnostic error), or in the organisation of care (i.e. inter-professional communication or co-ordination failures) [3, 4]. Safety incidents often refer to incidents that involve some form of harm for the patient. Safety incidents however can also include ‘precursors’, which have the potential to lead to harm if not prevented or managed appropriately [3]. For instance, adverse drug reactions/poisoning resulting from taking an incorrect drug or dose are viewed as ‘active safety incidents’, whereas errors in prescribing drugs which have the potential to lead to adverse drug reactions are considered ‘precursors of safety incidents’.

Although there have been significant improvements in delivery of care for long-term conditions [5], many quality improvement activities [6], clinical guidelines [7, 8] and innovations in service delivery [9] have focussed on the needs of patients with single long-term conditions. However, multimorbidity broadly defined as ‘the co-existence of two or more chronic conditions, where one is not necessarily more central than the others’ [10], is increasingly prevalent [11], and represents a major part of the workload of primary care [12].

Patients with multimorbidity are likely to be at risk from all types of safety incidents, due to a number of reasons. Individual patients with multimorbidity are more likely to have to manage complex medication and other management regimes [13], face difficult decisions about self-management and dealing with priorities among conditions and their management [14], and may not receive the quality of communication that is required to support them in the context of these demands [15]. The frequency and complexity of their interactions with health services may make them more vulnerable to failures of care delivery and co-ordination [16, 17]. Patients with multimorbidity are also likely to demonstrate characteristics which will further increase their vulnerability to safety incidents, such as poor health [2], advanced age [18], cognitive impairment [19], limited health literacy [20], and levels of depression and anxiety [21, 22]. Particularly, patients with multimorbidity with concurrent mental health conditions such as depression (referred to as ‘mental-physical’ multimorbidity hereafter) report lower quality of care compared with patients with physical long-term conditions only [23, 24]. It is likely that the time constrains and the tendency to prioritise the management of physical long-term conditions in primary care, adversely affects the quality of care delivered to patients with mental-physical multimorbidity [25–27]. Moreover, patients with mental-physical multimorbidity may have reduced capacity or motivation to self-care [28], receive less integrated care, and face more care co-ordination failures due to the complexity of their care needs [26, 29]. Therefore, patients with mental-physical multimorbidity might comprise a distinct, high-risk sub-group for safety incidents within the group of patients with multimorbidity.

Even though patients with multimorbidity may generally be at higher risk of safety incidents, there may be occasions where they face lower risk. Some measures of quality of care are higher in patients with multimorbidity [30]. This may reflect greater numbers of clinical encounters in such patients (with greater monitoring and opportunities to identify safety risks), or the development of self-management expertise in patients familiar with managing multiple, complex problems [31]. Our own research demonstrated that patient experience of aspects of care for long-term conditions did not differ markedly between patients with single or multiple conditions [32].

It is currently uncertain whether there are any specific groups of patients with multimorbidity who are more susceptible to patient safety incidents and whether there are any particular types of patient safety incidents that are more prevalent in patients with multimorbidity. This makes it difficult to design and embed interventions within existing care models to improve patient safety among people with multimorbidity.

We undertook a systematic review and evidence synthesis on multimorbidity and patient safety. This review sought to answer two research questions:

1. Are patients with multimorbidity more vulnerable to patient safety incidents?
2. Does the relationship between multimorbidity and patient safety vary across different types of multimorbidity and different types of patient safety outcomes?

## Methods

The methods and results for this review are reported in line with the Preferred Reporting Items for Systematic Reviews and Meta-Analyses (PRISMA) guidelines The completed PRISMA checklist is included in S1 PRISMA Checklist. [33].

### Search strategy

The following electronic bibliographic databases were searched for eligible papers: Medline, Embase and Cinahl (from inception until December 2013 and then updated to May 2015). Our search strategy included combinations of three key blocks of terms (*multimorbidity/ comorbidity*, *patient safety* and *primary care*) using a combination of Medical Subject Headings and text-words. (See the Medline search strategy in S1 Appendix. Database searches were supplemented by hand searches of reference lists of included papers. No previous reviews have been identified in the area. We excluded studies in languages other than English and studies in the grey literature.

### Eligibility criteria

Studies were eligible for inclusion in this review if they met the following criteria:

- Population: Adult patients (18 years or above) with two or more physical long-term conditions, or combinations of physical and mental long-term conditions. We included in the review patients with any physical long-term condition such as diabetes, chronic obstructive pulmonary disease, arthritis and hypertension. Mental long-term conditions mainly comprised mental health conditions such as depressive disorders and psychoses. We excluded studies which were solely based on patients with two or more mental health conditions (i.e. depression comorbid with schizophrenia) without concurrent physical long-term conditions. We also excluded studies based on patients with combinations of mental and substance use/alcohol use conditions.
- Design: Quantitative research design (case control, cross-sectional, retrospective or prospective cohort, or controlled trial).
- Setting: Primary care, the interface between primary and specialty care (e.g., emergency department), and studies in general population samples. We excluded studies conducted exclusively in specialist settings.
- Multimorbidity measure: We included studies which reported:
  - A measure of physical multimorbidity (based on simple count of long-term conditions or more complex multimorbidity indices) or combinations of two or more specific physical long-term conditions (such as comorbid diabetes with hypertension).
  - A measure of mental-physical multimorbidity, defined as combinations of physical and mental long-term conditions (such as diabetes and depression). Only studies which made an explicit distinction between physical and mental comorbidities were included in the “mental-physical multimorbidity” category. Studies which were not explicit about the inclusion of mental long-term conditions in the definition of multimorbidity were not included in this category.
- Patient safety incident: After reviewing the theoretical and empirical literature on patient safety, and after consultation with the expert patient safety researchers in the team, we distinguished two types of safety measures:
  - Active patient safety incidents i.e. measures of adverse outcomes or injuries stemming from the processes of health care [2], such as *adverse drug events* (resulting from wrong dose, drug-drug interactions) and *other adverse events* such as intervention complications, infections and care failures (i.e. pressure ulcers).
  - Precursors of patient safety incidents i.e. factors potentially leading to adverse outcomes or injuries [2]. This included *prescription errors* such as inappropriate prescribing, over or under utilisation of drugs, *medication non-adherence*, *diagnostic errors* such as wrong or delayed diagnosis and *poor quality of care* resulting from failure to adhere to established guidelines for care provision or from communication and co-ordination failures.
- Quantitative association between the multimorbidity measure and patient safety outcomes which was amenable to meta-analysis. We sought data that would allow the computation of an effect size (odds ratio) for the association of multimorbidity with patient safety outcomes. We sought data that would allow the computation of an effect size (odds ratio) for the association of multimorbidity with patient safety outcomes. We excluded studies that lacked data to compute an effect size of for the association between multimorbidity and patient safety outcome (i.e. only reported means without standard deviations or p-values).

### Study selection

Study selection was completed in two stages. Initially, the titles and abstracts of the identified studies were screened for eligibility by the first author. A subset of titles and abstracts (20%) were screened independently by a second reviewer (kappa coefficient = 0.78). Next, the full-texts of studies assessed as potentially relevant for the review were retrieved and checked against our inclusion and exclusion criteria. Forty percent of the full-text screening was completed by two researchers working independently. Any disagreements were resolved by discussion. Given the high inter-rater reliability (kappa coefficient = 0.85), the remaining full-text screening was completed by the first author.

### Data extraction

A data extraction form was devised in Microsoft Excel and piloted on five randomly selected studies. We extracted the following descriptive data: country, research design, population, recruitment method, research setting, participant characteristics (number, age, gender, long-term conditions), multimorbidity measure, patient safety outcomes and methodological quality. We also extracted quantitative data on the association between multimorbidity and patent safety. Thirty percent (n = 20 studies) of the data extraction was completed by 2 members of the research team working independently. No substantial disagreements were observed (kappa 0.91 across 2160 data points); the remainder of the data were extracted by one member and checked by a second.

### Methodological quality of the studies

The vast majority of the studies included in the review were observational studies (cross-sectional and cohort studies). As well as distinguishing these different designs, we also assessed methodological quality using criteria adapted from guidance on the assessment of observational studies [34]. Quality criteria were not used to exclude studies in the review. The quality appraisal included three key criteria:

1. Response rate or data capture among eligible patients of 70% or greater at baseline
2. Response rate or data capture of 70% or greater at follow-up (for prospective studies only)
3. Control for a minimum of 3 important confounding factors in the analysis which comprised a combination of demographic characteristics (age, gender) and clinical characteristics relevant to patient safety incidents (e.g. drug use/polypharmacy, contacts with health professionals and health services, disability levels).

These criteria have been previously used by members of our research group to assess the methodological quality of observational studies [35]. Studies were assigned a rating of 1 for each criterion met (maximum rating of 3). Substantial agreement between reviewers regarding methodological quality (kappa 0.89).

### Data synthesis

The primary outcome of this review was the effect of multimorbidity on patient safety outcomes (‘active patient safety incidents’ and ‘precursors of safety incidents’). From the available data we calculated odds ratios (ORs) together with the 95% confidence intervals from each study using the Comprehensive Meta-analysis (CMA) software [36]. ORs were typically computed from dichotomous data (number/rates of safety incidents), but continuous data (i.e. means) were also converted to ORs in CMA. CMA allows computation of ORs from several input parameters (dichotomous, continuous or both data types) including all eight methods proposed by the Cochrane Handbook [37], as well as additional methods proposed in the literature [38]. We chose ORs to pool the results because this was the most commonly reported estimate for effect in the individual studies, and because ORs are considered more appropriate for use across different research designs (including cross-sectional and case-control designs) compared with other estimates such as relative risks [39]. In this study, OR >1 indicates that multimorbidity is associated with increased risk for patient safety incidents, whereas OR <1 indicates that multimorbidity is associated with a lower risk for patient safety incidents. Across studies reporting adjusted and unadjusted models, we selected the model in which effect sizes were adjusted for potentially confounding variables to the maximum extent.

The I^2^-statistic was used to assess heterogeneity among studies. Conventionally, I^2^ values of 25%, 50%, and 75% indicate low, moderate, and high heterogeneity [40]. Subgroup analyses were performed to explore potential sources of heterogeneity of the relationship between multimorbidity and patient safety incidents (e.g. the effects of types of multimorbidity). In line with the Cochrane Handbook [41], we compared subgroups informally by comparing the magnitudes of effect within each. A sensitivity analysis was undertaken to evaluate the stability of the results after only the studies with higher ratings (as indicated by ratings on the 3 quality assessment criteria) were retained in the analysis. The possibility of publication bias was examined by inspecting the symmetry of the funnel plot and using Egger’s test [42].

In accordance with recommendations [43], across studies reporting multiple measures of the same safety incident (e.g. different measures of poor quality) or the same type of multimorbidity (e.g. effects of multiple types of physical comorbidities on adverse drug events) weighted average ORs were computed to ensure that each study contributed only one effect measure of each outcome to the meta-analysis. Where studies reported data on different types of safety incidents such as poor quality of care and prescription error (N = 3), or different types of multimorbidity including physical multimorbidity and mental-physical multimorbidity (N = 8), we also computed the mean of the comparisons for each study, and entered this aggregate score in the pooled analysis. However, in analyses in which there was no overlap of these comparisons (subgroup analyses examining the distinct effects of types of safety incidents or types of multimorbidity), each comparison was treated as a separate unit of analysis.

All meta-analyses were performed in STATA (version 12) using the metan command [44]. Funnel plots were constructed using the metafunnel command [45], and the Egger test was computed using the metabias command [46]. Random effects models were applied to calculate pooled ORs because of anticipated heterogeneity.

## Results

**Fig 1:**
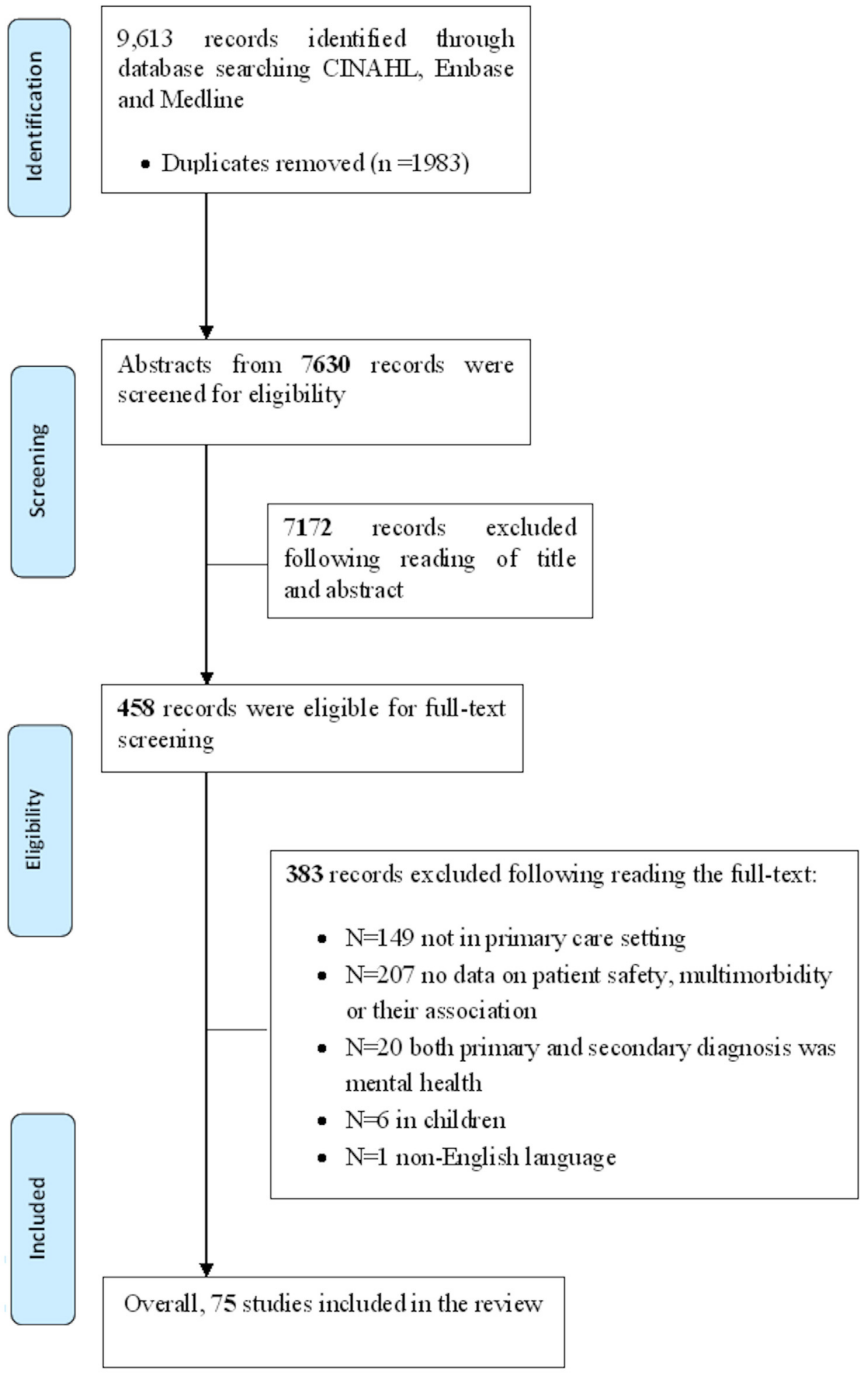
Flowchart of studies included in the review.

Overall, 7,630 titles and abstracts were screened for eligibility. Following screening, 75 studies (providing 86 relevant comparisons) met the inclusion criteria (Fig 1) [23, 24, 47–119]

### Characteristics of studies and populations

Key descriptive data for the studies included in the review are presented in Table 1. Details of data extracted from individual studies (study, population and outcomes) are provided in Table 2. Additional information about the population, outcomes and quality assessment is presented in S1 Table. Studies included patients with a wide range of ages, with the average around 60, and approximately equal gender representation. Most studies were based in primary care, and most common designs were retrospective cohorts and cross-sectional studies (see Table 2 for more details).

**Table 1:** Basic study descriptive data.

Category	Characteristics	N = 75 studies
**Study and population**	Sample size (range)	3,791,196 (40 to 1,265,434)
	Mean Age (range)	59 (38–80)
	% Male	52%
	**Country**	
	US	39 (52%)
	European	24 (32%)
	Other	12 (16%)
**Quality**	**Research design**	
	Cross-sectional	31 (41%)
	Prospective cohort	8 (11%)
	Retrospective cohort	32 (43%)
	Trial (randomized/non randomized)	1 (1%)
	Case control	3 (4%)
	**Methodological quality**	
	Response rate at baseline- 70% and over	29 (39%)
	Response rate at follow-up -70% and over	2 (3%)
	Control for confounding factors	47 (63%)
**Outcomes**	**Patient safety outcome**	**N = 86 comparisons**
	Active safety incidents	26 (30%)
	Adverse drug events	15 (17%)
	Other adverse events	11 (13%)
	Precursors of safety incidents	60 (70%)
	Quality of care	30 (35%)
	Prescription error	19 (22%)
	Medication non-adherence	6 (7%)
	Diagnostic error	5 (6%)
	**Multimorbidity**	**N = 86 comparisons**
	Mental-physical multimorbidity	20 (23%)
	Physical multimorbidity	66 (77%)

**Table 2:** Characteristics of studies, populations and outcomes included in the review.

Study ID	Country	Setting	Research design	N	Men (%)	Mean age (SD; Range)	Multimorbidity	Patient safety incident	Overall quality
Bae et al. 2008	US	General population	Cross-sectional	1,700	41%	18 years over	Physical multimorbidity	Poor quality of care	1
Barham et al. 2009	US	Primary care practices	Retrospective	1,701	41%	Range = 21–87	Physical multimorbidity	Poor quality of care	1
Beer et al. 2010	Australia	Primary care practices	Prospective	4,260	100%	M = 77; SD = 3.6; Range = 65–83	Physical multimorbidity	Prescription errors	0
Berger et al. 2009	Germany	Primary care practices	Retrospective	975	28%	M = 75.0; SD = 7.3; 65 and over	Mental-physical multimorbidity	Prescription errors	0
Bertomeu et al. 2009	Spain	Primary care and outpatient practices	Cross-sectional	2,767	72%	M = 67.5; SD = 11.4	Physical multimorbidity	Prescription errors	2
Blais et al. 2013	Canada	Charts of patients	Retrospective	1,200	n/a	M = 71.52	Physical multimorbidity	Other adverse events	2
Blecker et al. 2010	US	Medicaid claims data	Retrospective	1,801	31%	M = 58.7; SD = 9.4; Range = 21–62	Mental-physical multimorbidity	Prescription errors; Poor quality of care	1
Bont et al. 2007	Netherlands	Primary care practices	Retrospective	2,643	45%	M = 75; SD = 7.0; Range = 65–101	Physical multimorbidity	Prescription errors	1
Buja et al. 2014	Italy	Primary care practices	Retrospective	105,987	56%	16 and over	Physical multimorbidity	Poor quality of care	1
Cahir et al. 2013	Ireland	Primary care practices	Retrospective	931	47%	M = 78;SD = 5.4; Range: 70–98	Physical multimorbidity	Adverse drug events	2
Calderon et al. 2012	Spain	Primary care practices	Retrospective	79,089	44%	18 and over	Physical multimorbidity	Adverse drug events	2
Calvert et al. 2009	UK	Primary care practices	Retrospective	9,311	49%	M = 80.1; Range = 72.5–86.0	Physical multimorbidity	Prescription errors	2
Chen et al. 2011	Taiwan	Emergency department	Prospective	452	n/r	18 and over	Physical multimorbidity	Adverse drug events	2
Classen et al. 2007	US	General population	Retrospective	191	40%	Range = 60 over	Physical multimorbidity	Adverse drug events	1
Dalton et al. 2011	UK	Primary care practices	Cross-sectional	3,294	56%	M = 61; Range = 17 over	Physical multimorbidity	Poor quality of care	2
Davis et al. 2008	UK	General population	Retrospective	955	45%	M = 77.0; SD = 10.0	Physical multimorbidity	Prescription errors	0
Desai et al. 2005	US	Veterans Affairs health services	Cross-sectional	15,580	79%	M = 61.3; SD = 13.9	Physical multimorbidity	Diagnostic errors	1
Desai et al. 2006	US	Veterans Affairs health services	Cross-sectional	21,489	83%	n/r	Physical multimorbidity	Diagnostic errors	1
Drivenes et al. 2014	Norway	Primary Care Practicees	Cross-sectional	376	46%	Median = 62	Physical multimorbidity	Prescription errors	0
Druss et al. 2012	US	Medicaid claims data	Cross-sectional	113,505	84%	n/r	Mental-physical multimorbidity	Poor quality of care	1
Eguale et al. 2012	Canada	Primary care practices	Cross-sectional	50,823	n/r	n/r	Physical multimorbidity	Prescription errors	1
Fernandez et al. 2015	Spain	Primary care practices	Cross-sectional	1,214	79%	M = 66.4, SD = 9.7; R = 40 over	Physical multimorbidity	Diagnostic errors	2
Field et al. 2004	US	Primary care practices	Case-control	1,598	41%	M = 75.2; R = 65 over	Physical multimorbidity	Adverse drug events	0
Frigola et al. 2013	Netherlands	Primary care practices	Retrospective	7,173	41%	M = 76.3; SD = 10.7	Physical multimorbidity	Prescription errors	0
Ghembaza et al. 2014	Algeria	Primary care practices	Cross-sectional	453	24%	M = 62, SD = 1.16	Physical multimorbidity	Medication non-adherence	1
Goldberg et al. 2007	US	Primary care practices	Cross-sectional	300	59%	n/r	Mental-physical multimorbidity	Poor quality of care	2
Harman et al. 2004	US	General population	Retrospective	498	n/r	65 and over	Physical multimorbidity	Poor quality of care	1
Hayes et al. 2014	Canada	Primary care practices	Retrospective	187	62%	M = 44; Range = 35–55	Physical multimorbidity	Other adverse event	1
Hesse 2015	UK	Data from trials registered in Virtual International Stroke Trials Archive	Retrospective	5775	54%	M = 69.3, SD = 12.3	Physical multimorbidity	Other adverse events	1
Higashi et al. 2007	US	General population	Retrospective	7,680	48%	n/r	Physical multimorbidity	Poor quality of care	0
Ho et al. 2006	US	Primary care practices	Retrospective	11,532	51%	Range = 18 over	Physical multimorbidity	Medication non-adherence	0
Kanner et al. 2012	US	Primary care practices	Cross-sectional	188	32%	M = 39; SD = 11.7	Mental-physical multimorbidity	Adverse drug events	1
Katerndahl et al. 2012	US	Primary care practices	Cross-sectional	102	30%	M = 56.8; SD = 10.6	Mental-physical multimorbidity	Medication non-adherence; Poor quality of care	1
Ko et al. 2013	US	General population	Cross-sectional	40	60%	M = 58; SD = 13; Range: 24–88	Physical multimorbidity	Poor quality of care	0
Kontopantelis et al. 2013	UK	Primary care practices	Prospective	23,920	69%	M = 62.9; 18 and over	Physical multimorbidity	Poor quality of care	1
Krein et al. 2006	US	Veterans Affairs health services registries	Case control	36,546	97%	M = 58; SD = 12	Mental-physical multimorbidity	Poor quality of care	0
Lagomasino et al. 2005	US	Primary care practices	Cross-sectional	1,175	30%	M = 43.9; SD = 15.3; 18 and over	Physical multimorbidity	Prescription errors	2
Lin et al. 2013	US	Medicare claims records	Cross-sectional	19,863	70%	65 and over	Physical multimorbidity	Other adverse events	1
Lu et al. 2011	US	General population	Cross-sectional	11,910	40%	M = 51.1; SD = 16.3	Physical multimorbidity	Prescription errors	1
Mand et al. 2014	Germany	Primary care practices	Retrospective	24,619	63%	M = 75.7, SD = 7.8 Range = 65–107	Mental-physical multimorbidity/Physical multimorbidity	Adverse drug events	1
Marcum et al. 2012	US	Veterans Affairs Medical Centres	Retrospective	678	99%	M = 76.4; 65 and over	Physical multimorbidity	Adverse drug events	0
McGovern et al. 2013	UK	Primary Care	Retrospective	35,502	54%	M = 63.6; SD = 14.3	Physical multimorbidity	Other adverse events	1
Mensah et al. 2007	UK	Primary care practices	Cross-sectional	515	50%	M = 49, R = 78	Mental-physical multimorbidity	Adverse drug events	0
Mikuls et al. 2005	US	Primary care practices	Retrospective	708	72%	M = 61; SD = 15	Physical multimorbidity	Poor quality of care	1
Min et al. 2014	US	Primary care practices	Cross-sectional	644	67%	M = 80; over 70	Physical multimorbidity	Poor quality of care	2
Mira et al. 2014	Spain	Primary care practices	Cross-sectional	265	53%	M = 72.5; SD = 5.5; over 65	Physical multimorbidity	Adverse drug events	0
Nasser et al. 2009	Bahrain	Primary care practices	Cross-sectional	808	39%	20 and over	Mental-physical multimorbidity	Other adverse events	0
Nuyen et al. 2005	Netherlands	Primary care practices	Cross-sectional	191	28%	M = 45.4; SD = 14.1	Physical multimorbidity	Diagnostic errors	2
Obreli-Neto et al. 2012	Brazil	Primary care practices	Prospective	433	20%	M = 67; Range = 64–67	Physical multimorbidity	Adverse drug events	2
Parchman et al. 2005	US	Veterans Affairs Medical Centre	Cross-sectional	420	82%	n/r	Physical multimorbidity	Adverse drug events	1
Pawaskar et al. 2008	US	Primary care practices	Cross-sectional	5,487	40%	18 and over	Mental-physical multimorbidity; Physical multimorbidity	Prescription errors	1
Petersen et al. 2009	US	Veterans Affairs facilities	Prospective	141,609	n/r	M = 63.4; SD = 12.4	Physical multimorbidity	Poor quality of care	1
Pugh et al. 2005	US	Veterans Affairs outpatient facilities	Retrospective	1,265,434	98%	M = 73.5; SD = 5.6; 66 and over	Mental-physical multimorbidity; Physical multimorbidity	Prescription errors	1
Pugh, et al. 2010	US	Veterans Affairs and Medicare databases	Retrospective	9,682	98%	66 and over	Mental-physical multimorbidity; Physical multimorbidity	Adverse drug events	1
Reichard et al. 2012	US	Kansas Medicaid programme	Retrospective	9,532	35%	M = 53.5; 18 and over	Physical multimorbidity	Poor quality of care	1
Rigler 2004 et al.	US	Medicaid claims data	Retrospective	3,185	23%	65 and over	Physical multimorbidity	Prescription errors	2
Ruigomez et al. 2007	UK	UK General Practice Research Database	Prospective	906	48%	Range = 40–89	Physical multimorbidity	Other adverse events	2
Rupert, 2010 et al.	US	Primary care	Cross-sectional	295	55%	M = 62; SD = 14	Mental-physical multimorbidity; Physical multimorbidity	Poor quality of care	0
Schnitzer et al. 2012	Germany	Complains forwarded to Patient Commissioner in Germany	Cross-sectional	13,505	48%	n/r	Physical multimorbidity	Poor quality of care; Prescription errors	1
Shireman et al. 2010	US	Medicaid claims data	Retrospective	666	50%	M = 43.1; SD = 11.9	Physical multimorbidity	Poor quality of care	1
Simeone et al. 2012	US	Medical claims to MarketScan commercial database	Case-control	11,372	54%	M = 54.5; SD = 7.9	Physical multimorbidity	Other adverse events	0
Simpson et al. 2007	US	Primary care practices	Cross-sectional	2,198	38%	n/r	Physical multimorbidity	Poor quality of care	1
Sloane et al. 2004	US	Primary care practices	Cross-sectional	2,014	24%	65 and over	Physical multimorbidity	Prescription errors	2
Streit et al. 2014	Sweden	Primary care practices	Retrospective	1,002	56%	M = 65; 50 to 80	Physical multimorbidity	Poor quality of care	1
Thorpe et al. 2012	US	Centres for Medicare and Medicaid Services	Retrospective	288,805	38.20%	65 and over	Mental-physical multimorbidity; Physical multimorbidity	Poor quality of care	0
Tomio et al. 2010	Japan	National Health Insurance claims data	Retrospective	636	49%	M = 72.7; SD = 9.2	Mental-physical multimorbidity; Physical multimorbidity	Poor quality of care	1
Tsang et al. 2013	UK	General Practice Research Database	Cross-sectional	74,763	48%	n/r	Physical multimorbidity	Other adverse events	2
van Dijk et al. 2007	Netherlands	Dutch general practice registration database	Retrospective	21,524	n/r	n/r	Physical multimorbidity	Medication non-adherence	2
Weisman et al. 2007	US	Primary care practices	Controlled, randomized, double-blinded trial	535	78%	M = 59.3, Range = 23–85	Physical multimorbidity	Other adverse events	1
Whooley et al. 2008	US	Primary care practices	Prospective	1,017	41%	M = 63; SD = 12	Mental-physical multimorbidity	Medication non-adherence	1
Wolff et al. 2002	US	Medicare beneficiaries database	Cross-sectional	1,217,103	39%	M = 75.4; 65 and over	Physical multimorbidity	Other adverse events	1
Wong et al. 2015	Canada	National Ambulatory Care Reporting System database	Retrospective	56,767	53%	M = 66, SD = 15	Physical multimorbidity; Mental-physical multimorbidity	Poor quality of care	1
Wong et al. 2011	China	Primary care clinics	Retrospective	12,875	44%	65 and over	Physical multimorbidity	Medication non-adherence	1
Woodard et al. 2012	US	Veterans affairs medical centres	Prospective	35,872	n/r	M = 58.7	Physical multimorbidity	Poor quality of care	0
Zwar et al. 2011	Australia	Primary care practices	Cross-sectional	445	49%	Range = 40–80	Physical multimorbidity	Diagnostic errors	0

*Note*: n/r = not reported, M = mean, R = range, SD = standard deviation

### Characteristics of multimorbidity measures and patient safety outcomes

Multimorbidity was assessed using a wide range of methods. Sixty-six comparisons reported a measure of physical multimorbidity based on index tools for multimorbidity such as the Charlson index [120], simple counts of diseases or listing specific physical comorbidities among long-term conditions. The remaining comparisons examined the mental-physical comorbidity (n = 20, where mental health condition was mainly depression). Eight studies reported an analysis of both physical multimorbidity and mental-physical multimorbidity.

‘Active patient safety incidents’ were reported in 26 comparisons and comprised the following outcomes:

1. adverse drug events including drug-drug interactions and drug side effects
2. adverse non-drug related medical events such as medical complications and adverse medical outcomes associated with care delivery

‘Precursors of patient safety incidents’ were reported in 60 comparisons and comprised the following outcomes:

1. poor quality of care (non-adherence to guidelines and quality indicators)
2. prescription errors such as over or underuse of drugs or inappropriate prescribing
3. medication non-adherence
4. diagnostic errors

### Methodological quality characteristics

In terms of the individual quality criteria, 29 studies reported a response rate of 70% or greater, and 47 studies adjusted for confounders in the analyses. Only 2 of the 8 prospective studies reported response rates at follow-up. Nineteen (25%) studies met at least 2 of the 3 quality criteria whereas only two studies met all three criteria (see Tables 1 and 2).

### Meta-analysis: Active safety incidents and multimorbidity

**Fig 2:**
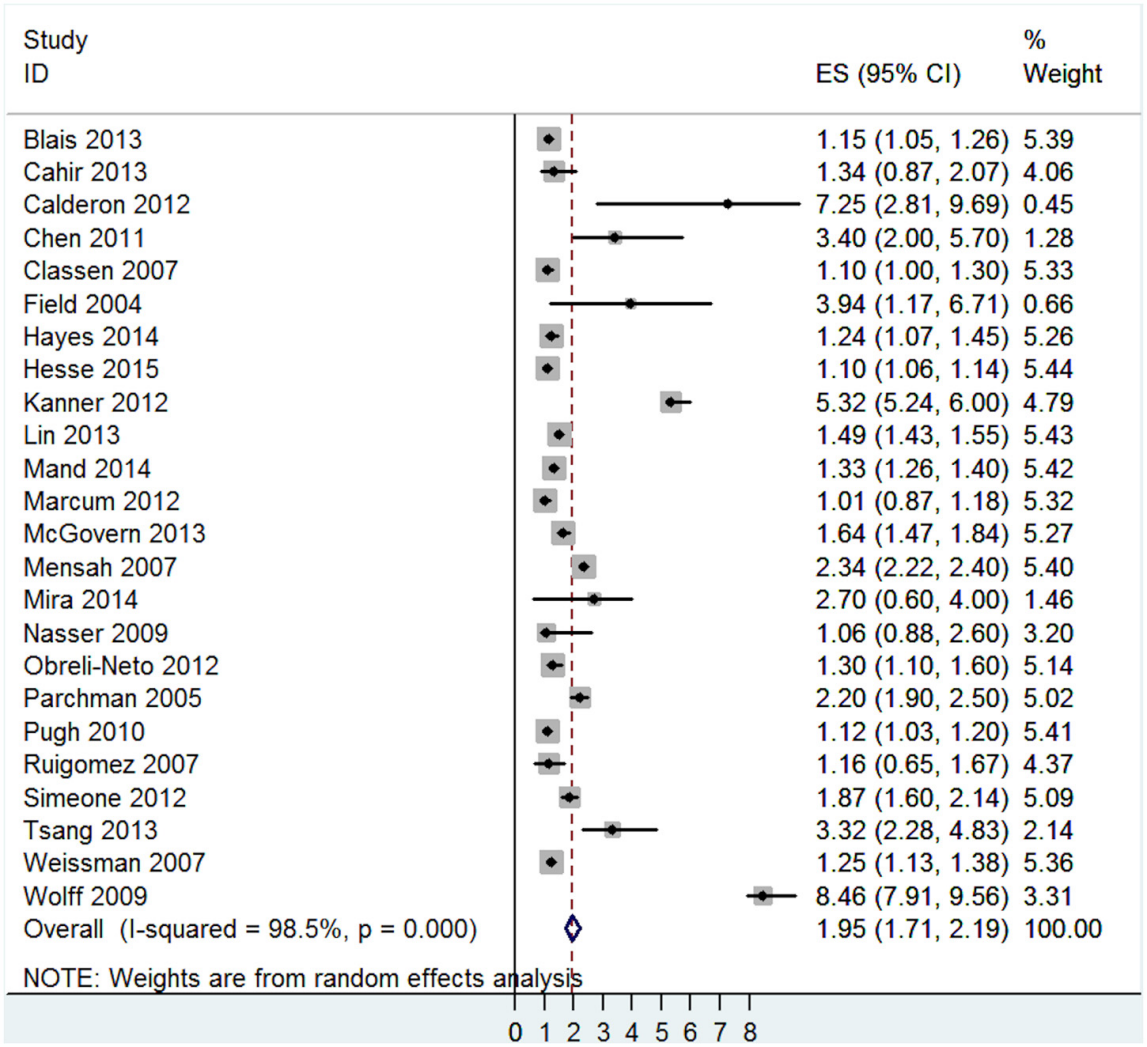
Main analysis of the association between active safety incidents and multimorbidity.

The pooled effect indicated that multimorbidity was associated with a significantly increased risk for active safety incidents, but outcomes exhibited high heterogeneity (OR = 1.95, 95% CI = 1.75 to 2.19, I^2^ = 98.5%, p<0.001- Fig 2). No studies reported that multimorbidity was related to significantly fewer active safety incidents.

**Fig 3:**
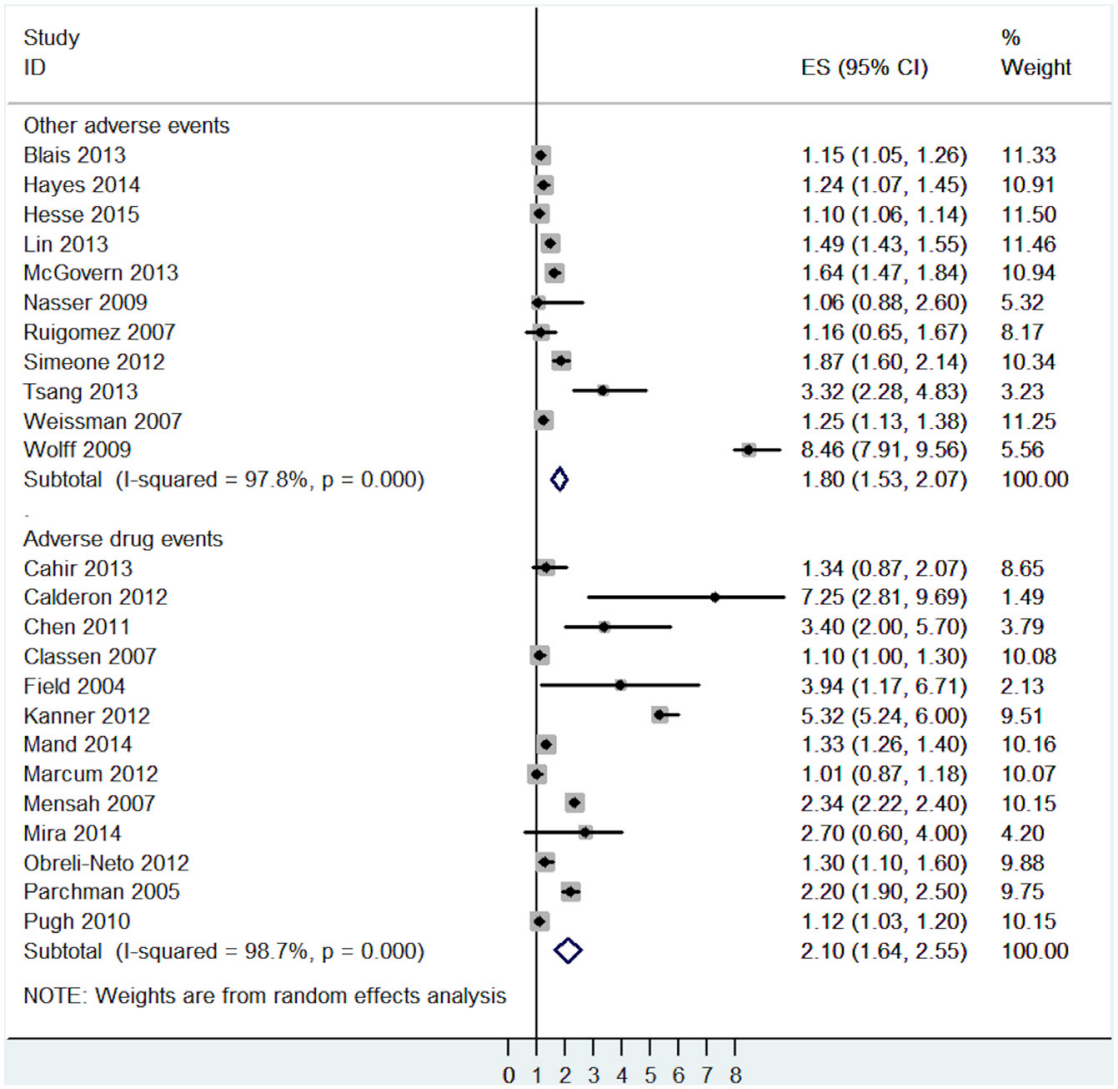
Subgroup analysis of the association between active safety incidents and multimorbidity analysed by different types of active safety incidents.

The effects of multimorbidity were broadly similar on the two types of active safety incidents: adverse drug events (OR = 2.10, 95% CI = 1.64 to 2.55, I^2^ = 98.7%, p<0.001) and other adverse events (OR = 1.80, 95% CI = 1.53 to 2.07, I^2^ = 97.8%, p<0.001—Fig 3).

**Fig 4:**
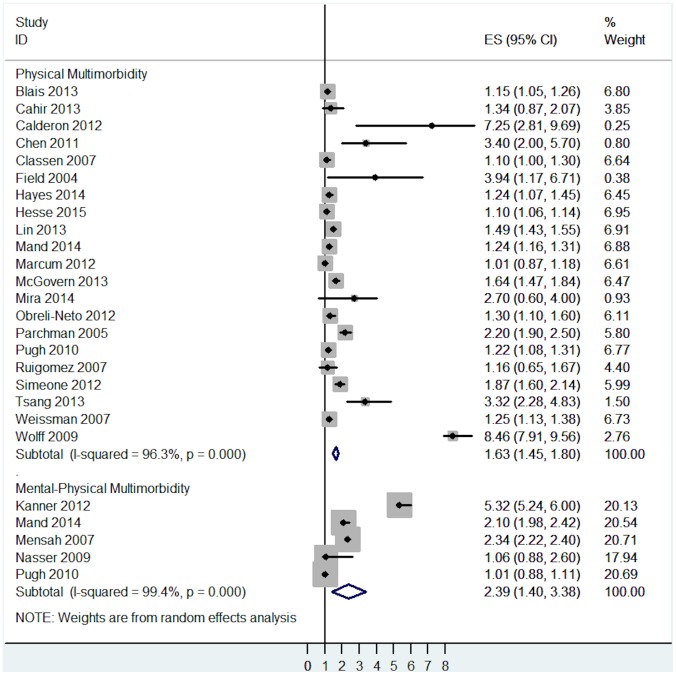
Subgroup analysis of the association between active safety incidents and multimorbidity analysed by different types of multimorbidity.

Moreover, mental-physical multimorbidity was associated with a higher risk for active safety incidents when compared with physical multimorbidity only (OR = 2.39, 95% CI = 1.40 to 3.38, I^2^ = 99.4%, p<0.001 and OR = 1.63, 95% CI = 1.45 to 1.80, I^2^ = 96.3%, respectively—Fig 4).

### Meta-analysis: Precursors of safety incidents and multimorbidity

A mixed pattern of findings was observed for precursors of safety incidents. The pooled effect size indicated that multimorbidity was associated with marginally increased risk for precursors of safety incidents, while the heterogeneity was high (OR = 1.16, 95% CI = 1.04 to 1.28, I^2^ = 98.6%, p<0.001- data not shown).

A notable proportion of the comparisons (n = 25) reported that multimorbidity was significantly associated with a higher risk of precursors of safety incidents, whereas a smaller but considerable proportion of the comparisons (n = 15) reported that multimorbidity was associated with a lower risk of precursors (mainly with quality of care).

There was some evidence that the risk for precursors of safety incidents was moderated by types of multimorbidity. Mental-physical multimorbidity was associated with increased risk for precursors of safety incidents (OR = 1.69, 95% CI = 1.36 to 2.03, I^2^ = 99.0%, p<0.001- data not shown) whereas no association was found between physical multimorbidity and precursors of safety incidents (OR = 1.02, 95% CI = 0.90 to 1.13, I^2^ = 98.0%, p<0.001-data not shown). However, heterogeneity remained high, and the results for each subtype of precursors of safety incidents (and the effects of types of multimorbidity on each outcome) are presented separately below.

#### Poor quality of care

**Fig 5:**
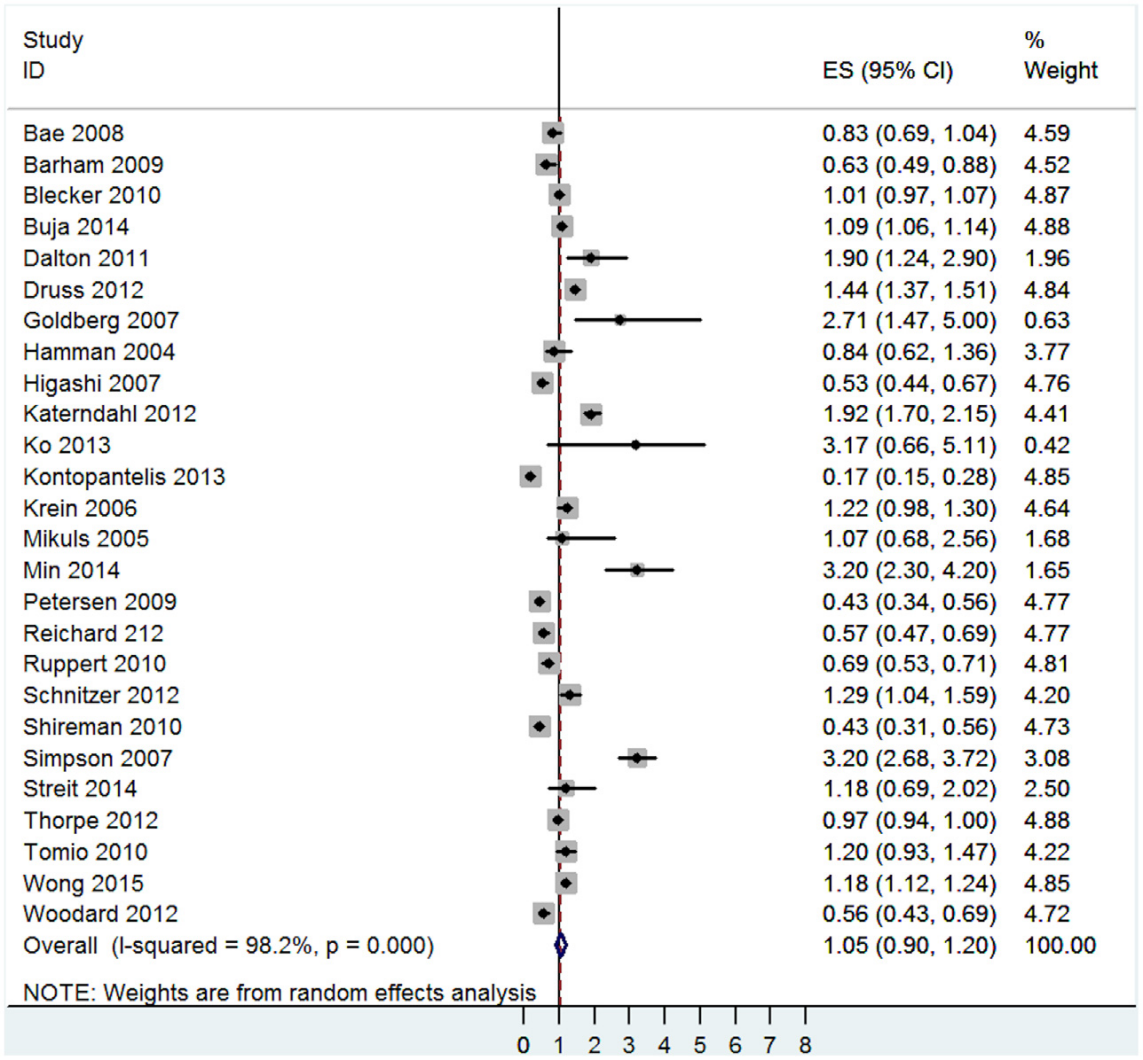
Main analysis of the association between poor quality of care and multimorbidity.

The pooled effect size indicated no association between poor quality of care and multimorbidity (OR = 1.05, 95% CI = 0.90 to 1.20, I^2^ = 98.2%, p<0.001—Fig 5). Heterogeneity was high, with 9 comparisons indicating that multimorbidity was associated with a higher risk for poor quality of care whereas 8 comparisons indicated that people with multimorbidity were less likely to experience poor quality of care.

**Fig 6:**
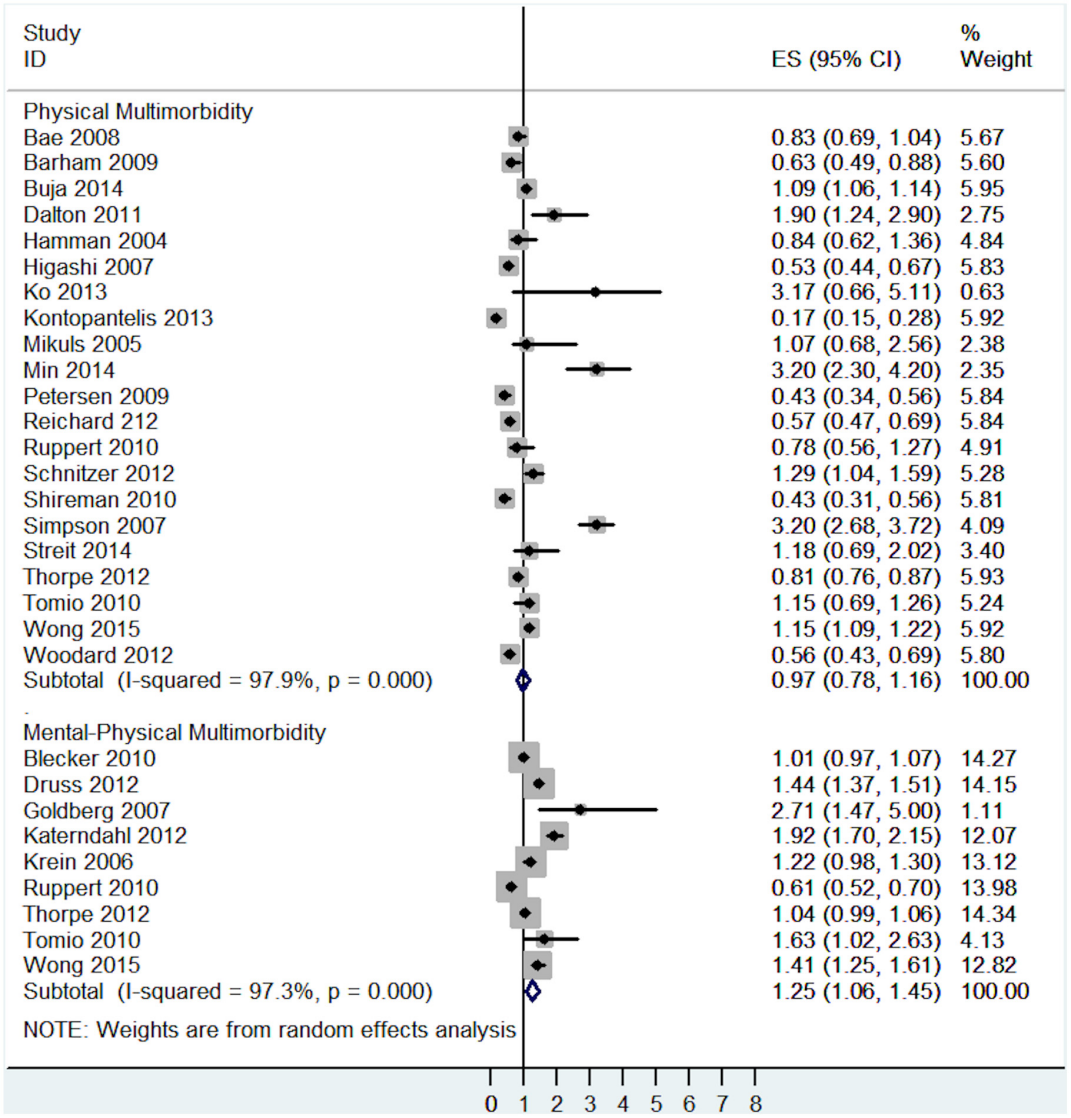
Subgroup analysis of the association between poor quality of care and multimorbidity analysed by different types of multimorbidity.

The risk for poor quality of care varied across types of multimorbidity. Mental-physical multimorbidity was associated with poorer quality of care (OR = 1.25, 95% CI = 1.06 to 1.45, I^2^ = 97.3%, p<0.001- Fig 6), whereas the effects of physical multimorbidity on quality of care were non-significant (OR = 0.97, 95% CI = 0.78 to 1.16, I^2^ = 97.9%, p<0.001- Fig 6).

#### Prescription errors

**Fig 7:**
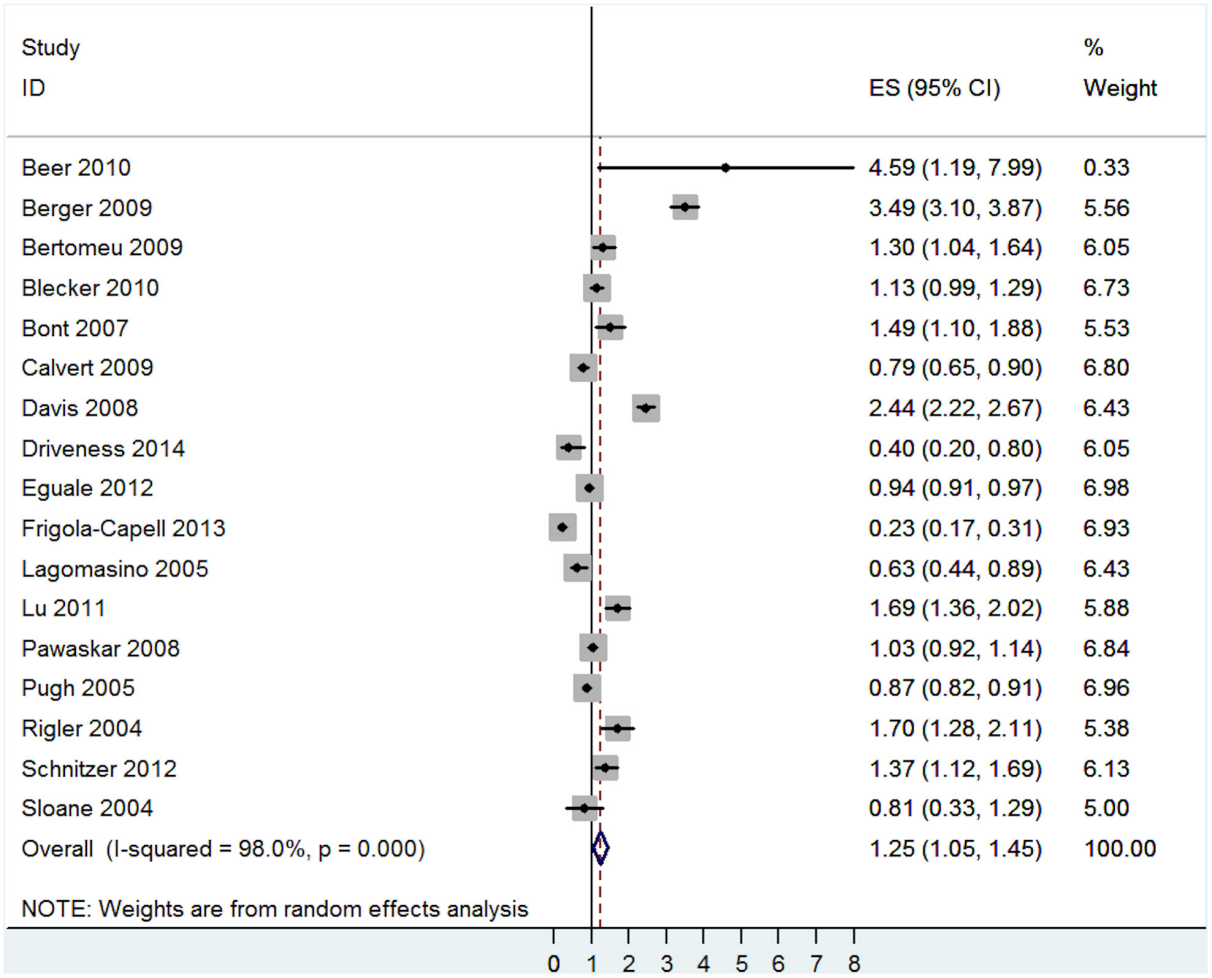
Main analysis of the association between prescription error and multimorbidity.

The pooled effect size was significant (OR = 1.25, 95% CI = 1.05 to 1.45, I^2^ = 98.0%, p<0.001—Fig 7) indicating that multimorbidity is associated with heightened risk for prescription errors. However, the results showed high levels of heterogeneity, with studies showing both positive and negative associations between multimorbidity and prescription errors.

**Fig 8:**
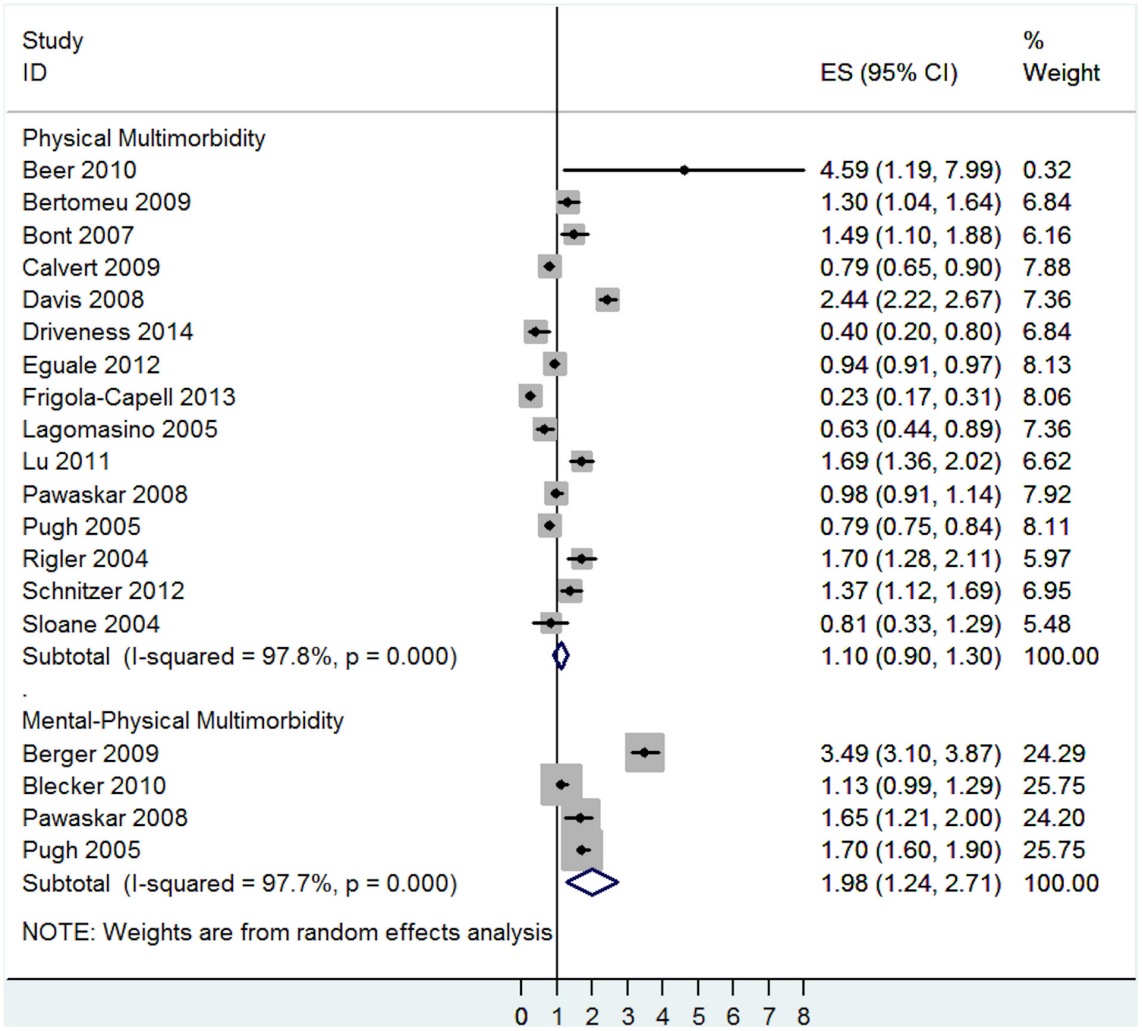
Subgroup analysis of the association between prescription error and multimorbidity analysed by different types of multimorbidity.

There was some evidence that the risk for prescription errors varied across types of multimorbidity. Mental-physical multimorbidity was associated with higher risk for prescription errors (OR = 1.98, 95% CI = 1.24 to 2.71, I^2^ = 97.7%, p<0.001—Fig 8). In contrast, no association was found between physical multimorbidity and prescription errors (OR = 1.10, 95% CI = 0.90 to 1.30, I^2^ = 97.8%, p<0.001—Fig 8).

#### Medication non-adherence

**Fig 9:**
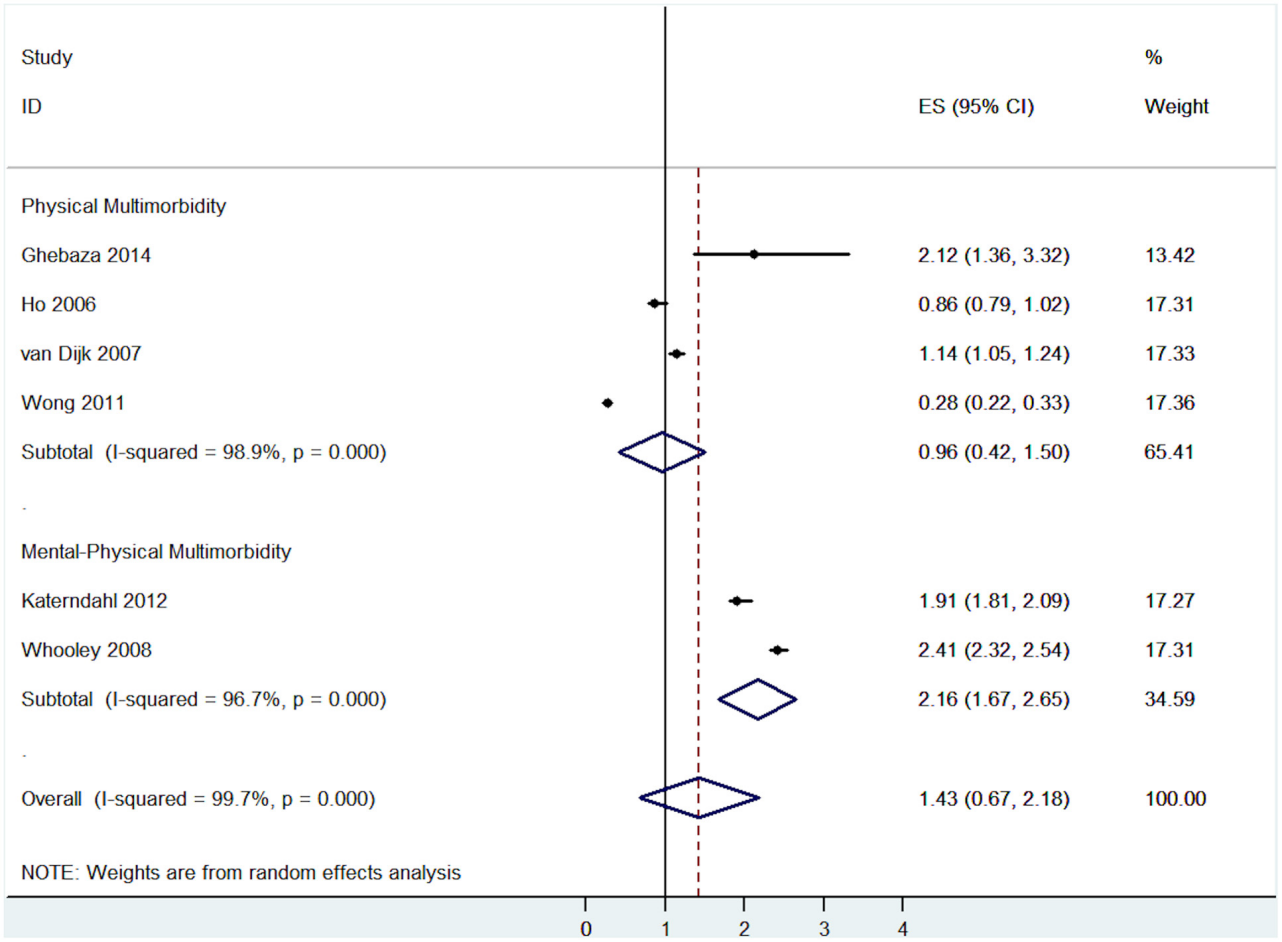
Association between medication non-adherence and multimorbidity analysed by different types of multimorbidity.

Six studies examined the link between medication non-adherence and multimorbidity. The pooled effect size indicated that multimorbidity had no effect on medication non-adherence (OR = 1.43, 95% CI = 0.67 to 2.18, I^2^ = 99.7%, p<0.001—Fig 9).

The risk for medication non-adherence varied across types of multimorbidity, but the number of studies was small. Studies on mental-physical multimorbidity reported a higher risk of medication non-adherence (OR = 2.16, 95% CI = 1.67 to 2.65, I^2^ = 96.7%, p<0.001- Fig 9), whereas the effects of physical multimorbidity on medication non-adherence were non-significant (OR = 0.96, 95% CI = 0.42 to 1.50, I^2^ = 98.9%, p<0.001- Fig 9).

#### Diagnostic errors

**Fig 10:**
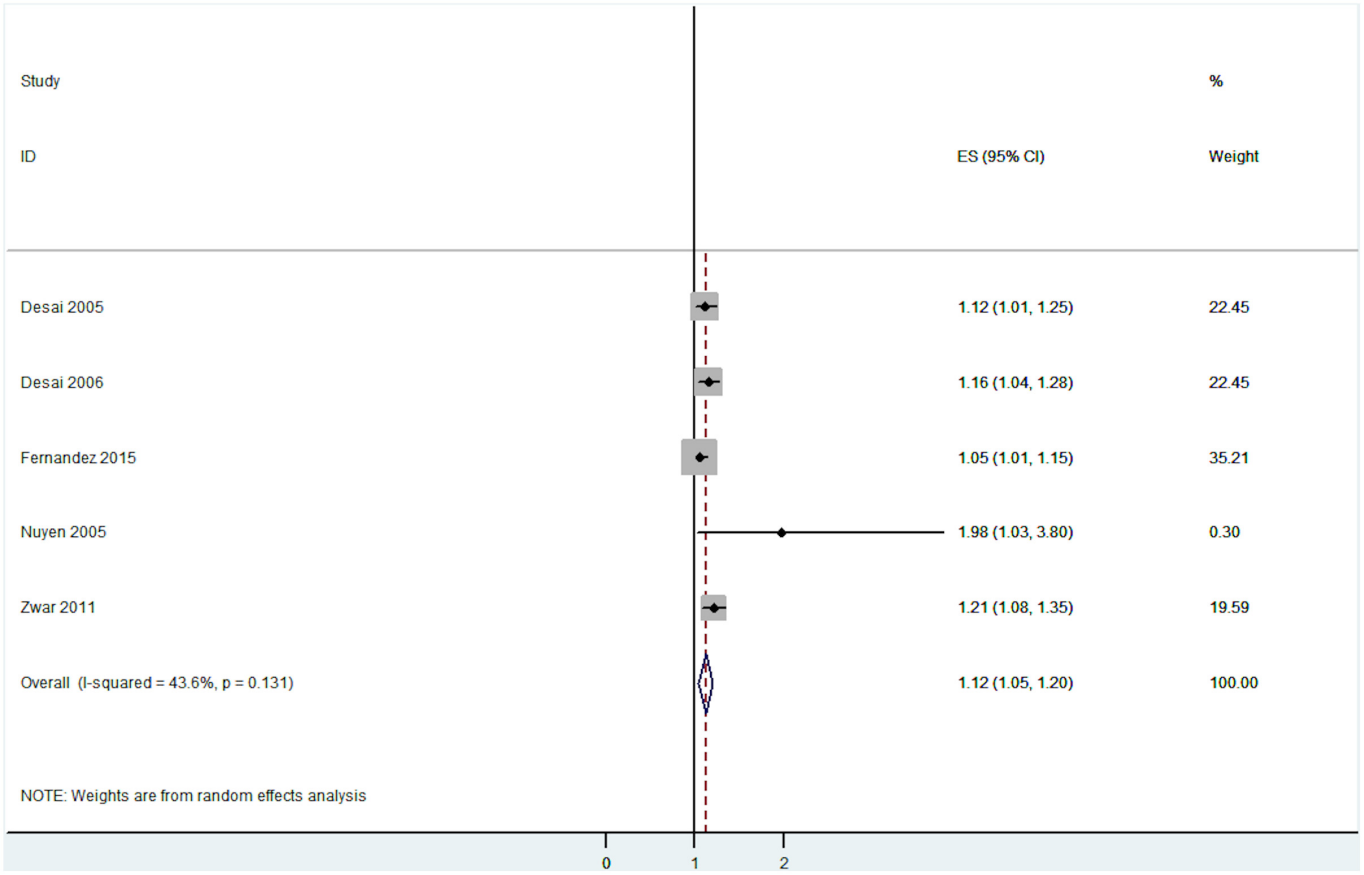
Association between diagnostic error and multimorbidity.

All five studies included in this category measured physical multimorbidity and demonstrated that it was associated with an increased risk of diagnostic errors. The pooled effect size was significant (OR = 1.12, 95% CI = 1.05 to 1.20, I^2^ = 43.6%, p = 0.131—Fig 10).

### Sensitivity analyses

**Fig 11:**
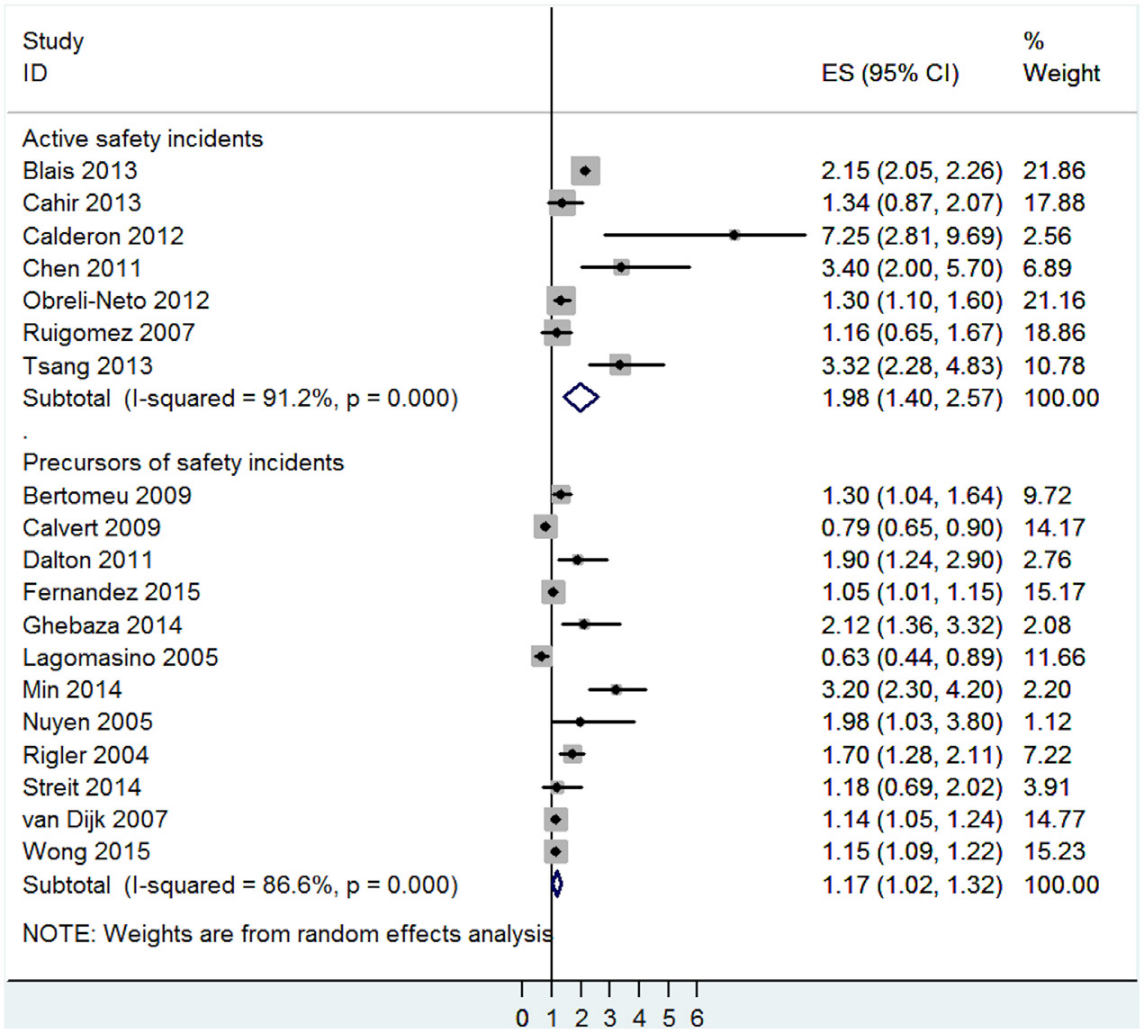
Sensitivity analysis examining the effects of multimorbidity on active safety incidents and precursors of safety incidents across studies with superior methodological quality scores.

The main findings for active safety incidents and precursors of safety incidents did not differ when only the 19 studies with sufficient methodological quality scores (meeting 2 out of 3 quality criteria of our protocol) were retained in the analyses. Multimorbidity was associated with significant increases in the risk for active safety incidents (OR = 1.98, 95% CI = 1.40 to 2.57, I^2^ = 91.2%, p<0.001) and marginal increases in the risk for precursors of safety incidents (OR = 1.17, 95% CI = 1.02 to 1.32, I^2^ = 86.6%, p<0.001- Fig 11).

### Publication bias

**Fig 12:**
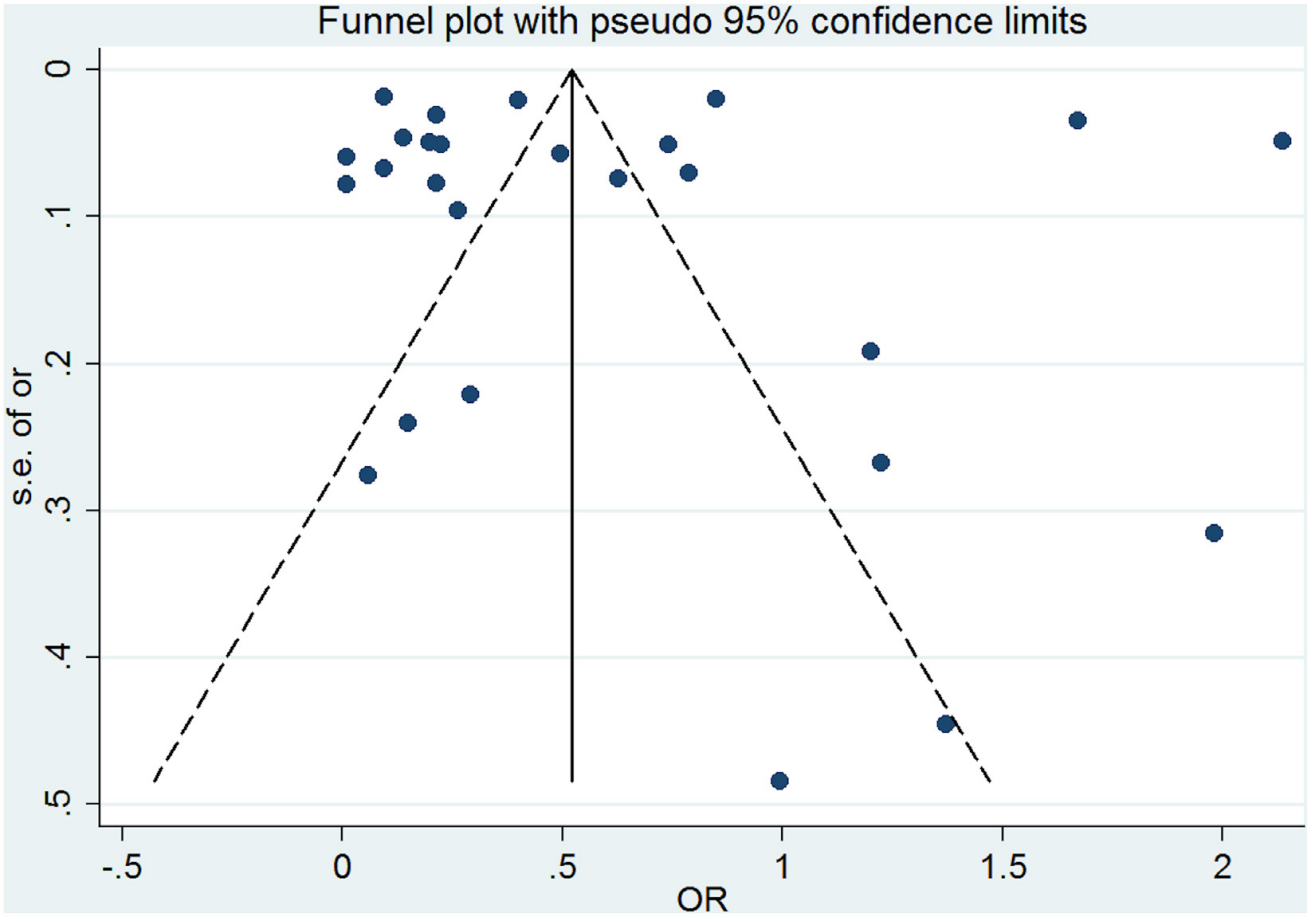
Funnel plot for studies examining the link between multimorbidity and active safety incidents.

**Fig 13:**
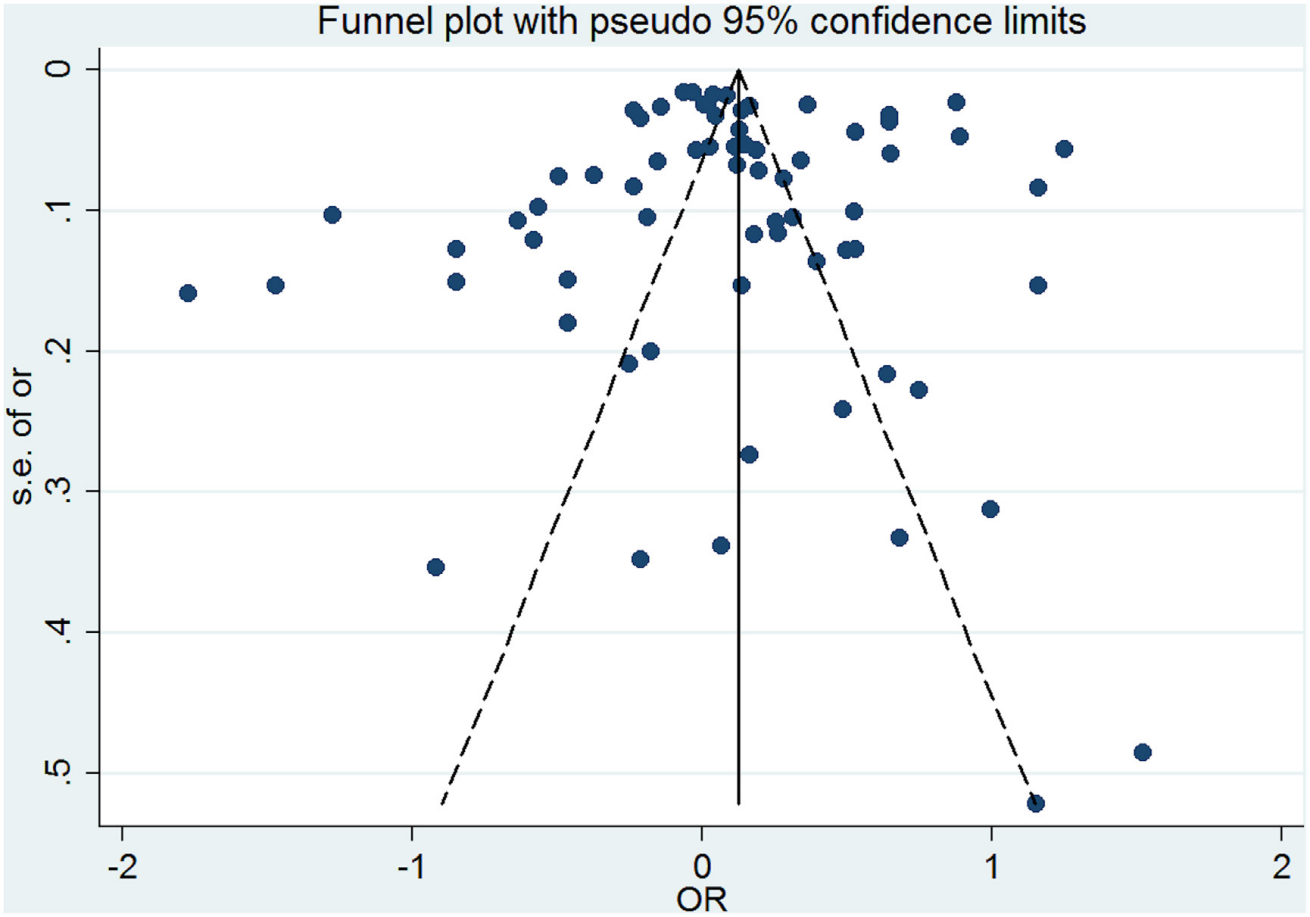
Funnel plot for studies examining the link between multimorbidity and precursors of safety incidents.

The Egger test was significant for active safety incidents indicating that the results in this category might be influenced by publication bias (regression intercept = -7.32, SE = 4.24, p = 0.05- Fig 12). No funnel plot asymmetry was identified and the Egger test was non-significant for studies examining precursors of safety incidents (regression intercept = -2.09, SE = 2.05, p = 0.312- Fig 13).

## Discussion

### Summary of the findings

The main aim of this study was to provide the first systematic review of the relationship between multimorbidity and patient safety outcomes. This review found that the relationship between multimorbidity and patient safety outcomes in primary care is complex, with high levels of variability, and may be influenced by different types of safety outcomes and types of multimorbidity. Both mental-physical multimorbidity and physical multimorbidity were associated with higher risk for active safety incidents (such as adverse drug events and medical complications), whereas mental-physical comorbidity (mainly depression) was associated with an increased risk for both active safety incidents and precursors of safety incidents (such as lower quality of care, prescription errors and medication non-adherence). In contrast, physical multimorbidity alone did not increase the risk of precursors of safety incidents, and in some cases was associated with a lower risk for safety failures (e.g. a trend was observed for physical multimorbidity to be associated with better quality of care).

### Strengths and limitations

This study has several strengths. This is the first systematic review to provide a comprehensive synthesis of the association between multimorbidity and patient safety outcomes, with a focus on primary care settings, where most of the healthcare for multimorbidity is delivered [12]. This review was performed and reported according to PRISMA guidance [33]. The searches were designed to be comprehensive and the eligibility criteria were broad, to ensure we incorporated all the evidence in the area.

This study also has important limitations. The review comprises of studies with heterogeneous populations and outcomes. In particular, a wide range of patient safety incidents were included in this review, and even outcomes included under the same subcategory (i.e. precursors) exhibited substantial variation. For example, the ‘poor quality of care’ category included a variety of incidents including problems in accessing care or receiving inappropriate care, and problems with preventative care. Similarly, different types of multimorbidity were reported across the studies.

We endeavoured to account for the large heterogeneity by applying random effects models, to adjust for between-study variations, and by undertaking subgroup analyses to explore key factors that may account for variation. We only explored the impact of basic sources of heterogeneity (e.g. different types of safety incidents and types of multimorbidity—broadly split into physical/ mental-physical), because multiple subgroup analyses inflate the probability of finding false results [41]. However, there are a large number of other factors which could explain the variability in the results of the subgroup analyses. An important factor may be the combinations of conditions which are likely to be included within each of our multimorbidity subgroups. More work may be needed to look at more precise combinations of diseases, or their clustering, which may affect safety outcomes. However, for this to be accomplished, individual studies must clear and consistent about the conditions which are included [121].

There is an argument that meta-analysis is inappropriate in the context of high levels of clinical, methodological and statistical heterogeneity [122], and the data may be more suited to a narrative synthesis. However, such syntheses are difficult to interpret when many studies are included. We adopted meta-analysis to allow us to compare results across studies, to examine the consistency of effects and explore variables that might account for inconsistency. These results may be at least as important as the pooled estimates we present [123].

Grey literature was not included in this review, which may have introduced study selection bias. We excluded grey literature based on evidence suggesting that the quality of research contained in the grey literature is lower and more difficult to appraise compared with research contained in journal articles [124]. Visual inspection of the funnel plot and Egger test did not identify evidence of publication bias for studies examining precursors of safety incidents, although publication bias was a possible risk for studies examining active safety incidents.

The large number of studies included in this review did not allow for the involvement of two independent researchers across all data screening and extraction, but reliability tests were performed which indicated high levels of inter-rater agreement. A less comprehensive quality assessment was also undertaken to account for the large number of studies and their variability. Despite this, the assessment of the methodological quality of the studies was designed to allow comparability across multiple different study designs, and were selected based on evidence suggesting that they reflect important quality aspects of observational studies [34]. The design of the original studies (mostly cross-sectional and retrospective) obviously imposes limits on our ability to establish causal links between multimorbidity and patient safety and the mechanisms that underpin these links.

### Implication for research, policy and practice

Our ability to draw inferences and offer recommendations is significantly hampered by the heterogeneity and inconsistent reporting of outcomes across the studies. Examining the link between multimorbidity and patient safety was usually a secondary aim of the studies. To improve patient safety in multimorbidity we need more primary research which explicitly addresses the relationship between patient safety precursors and incidents in people with multimorbidity. Specifically we need research that examines the mechanism by which multimorbidity affects patient safety. Large prospective studies which can establish temporal relationships are clearly needed to elucidate the relationship between multimorbidity and patient safety, and nested qualitative work may be useful to further illuminate how safety failures come about. Understanding mechanisms is crucial to guide the design of interventions to ameliorate threats to safety in people with multimorbidity. This may need to focus especially on the role of mental health, which the review suggests is an important drivers of safety outcomes.

Additionally, the development of common terminology, measures and reporting is a priority to ensure that future syntheses are not hampered by inconsistent presentation of data [125]. A recent systematic review which focused on the effects of comorbidity on benefits of treatment for long-term conditions encountered similar problems in terms of terminology, low methodological quality and lack of studies with a primary focus on the benefits and harms related to the health care of people with multimorbidity [99].

The range of safety outcomes reported was limited. For example, the frequency and complexity of healthcare needs and interactions of people with multimorbidity suggests that communication failures may be a key precursor for safety incidents in patients with multimorbidity [126, 127]. However, we found no studies examining the effects of multimorbidity on other types of safety incidents, such as patient-health professional or inter-professional communication or co-ordination of care. Work in these areas should be a priority.

At present there is limited evidence about the impact of interventions in patients with multimorbidity [128]. We only identified one trial which examined the effectiveness of a medication review and educational intervention in reducing hospitalisations due to adverse drug events in a high risk elderly population. This study showed that patients with severe multimorbidity (having 5 or more diseases) were significantly more likely to benefit from the intervention compared with patients with low or moderate multimorbidity [129]. This finding is encouraging, because it suggests that safety failures are amenable to intervention in patients with high levels of multimorbidity, and that the effects may be greater in those with greater numbers of conditions, a finding which has also been reported in treatment trials [130]. Individually-tailored care models which place emphasis on engaging patients with multimorbidity in their care may be a fruitful approach for reducing safety failures [127, 131, 132].

### Conclusion

This is the first systematic review of the association between multimorbidity and patient safety incidents in primary care. Although the patterns of association are complex, a key finding was that patients with multiple long-term conditions and patients with mental-physical comorbidity are at heightened risk for safety failures in some cases. The current evidence on the link between patient safety and multimorbidity is limited in scope and quality. Research is needed to improve the evidence base, to ensure that clinical practice, service organisation and health policy can promote safety in this group of patients.

## Supporting Information

S1 PRISMA Checklist PRISMA Checklist. (PDF)

S1 Appendix Medline search strategy. (PDF)

S1 Table Details of population, outcomes and study quality ratings. (PDF)
