# Supplementary material for: Multimorbidity and Patient Safety Incidents in Primary Care: A Systematic Review and Meta-Analysis
Source: PLoS One. 2015 Aug 28;10(8):e0135947. doi: 10.1371/journal.pone.0135947 (PMC4552710; doi:10.1371/journal.pone.0135947)
Supplement: S1 Appendix — (PDF) [file pone.0135947.s002.pdf]

S2 Appendix. Medline search strategy

. 1 Family Practice/

. 2 Primary Health Care/

. 3 Physicians, Family/

. 4 Community Health Services/

. 5 Hospitals/

. 6 Community Health Nursing/

. 7 Community Mental Health Services/

. 8 Community Pharmacy Services/

. 9 Home Care Services/

. 10 Community Mental Health Centers/

. 11 family pract$.tw.

. 12 general practice$.tw.

. 13 (communityadj (based or resident)).tw.

. 14 general population.tw.

. 15 nursing home$.tw.

. 16 community care.tw.

. 17 family medicine.tw.

. 18 family physician$.tw.

. 19 primary care.tw.

. 20 (primary health care or primary healthcare).tw.

. 21 family doctor$.tw.

. 22 primary medical care.tw.

. 23 general physician$.tw.

1

. 24 general practitioner$.tw.

. 25 primary care practitioner$.tw.

. 26 (communityadj (health or healthcare or health care)).tw.

. 27 primary healthcare team$.tw.

. 28 primary health care team$.tw.

. 29 primary medical care team$.tw.

. 30 practice nurse$.tw.

. 31 practice manager$.tw.

. 32 Hospitals.tw.

. 33 (practitioner$ adj3 special interest$).tw.

. 34 (primary care or primary health care or primary healthcare or general practice or family practice or family medicine).nw,in.

. 35 or/1-34

. 36 Comorbidity/

. 37 comorbid$.tw.

. 38 multimorbid$.tw.

. 39 multi-morbid$.tw.

. 40 polypathy.tw.

. 41 polypathology.tw.

. 42 polypathologies.tw.

. 43 polymorbid$.tw.

. 44 multiple pathology.tw.

. 45 multiple pathologies.tw.

. 46 ((co-occurrence or cooccurence or co-occurring or cooccuring or concurrent) adj2 (disease$ or illness$ or condition$ or disorder$)).tw.

2

. 47 (multiple$ adj5 (chronic disease$ or chronic illness$ or chronic condition$)).tw.

. 48 cumulative illness.tw.

. 49 or/36-48

. 50 35 and 49

. 51 exp Patient Safety/

. 52 exp Safety Management/

. 53 exp Iatrogenic Disease/

. 54 exp Medical Errors/

. 55 exp Malpractice/ "quality of health care"/ or "outcome and process assessment (health care)"/ or quality

. 56 assurance, health care/ or quality improvement/ or quality indicators, health care/ or "health care quality, access, and

evaluation"/

. 57 patient safety$.tw.

. 58 safety culture$.tw.

. 59 (safe$ adj2 (practice$ or manage$)).tw.

. 60 iatrogenic.tw.

. 61 malpractice.tw.

. 62 harm$.tw.

. 63 negligence.tw.

. 64 negligent.tw.

. 65 near miss$.tw.

. 66 human error$.tw.

. 67 ((service$ or system$ or communication$ or organisation$ or organization$) adj2 (weak$ or fail$)).tw.

. 68 (latent adj1 (threat$ or cause$ or fail$)).tw.

. 69 exp Adverse Drug Reaction Reporting Systems/

3

. 70 *Accident Prevention/

. 71 exp Self-Injurious Behavior/

. 72 ((adverse or avoidable or preventable or unsafe or safet$) adj2 (event$ or outcome$ or complication$ or death$ or effect$ or reaction$ or accident$ or injur$)).tw.

. 73 ((medica$ or diagnostic or therapeautic or administration or dispensing or prescri$) adj2 (error$ or mistake$ or fault$)).tw.

. 74 (patient$ adj2 (risk$ or incident$ or accident$)).tw.

. 75 incident report$.mp.

. 76 *Risk Management/

. 77 or/51-76

. 78 50 and 77
