# Supplementary material for: Multimorbidity and Patient Safety Incidents in Primary Care: A Systematic Review and Meta-Analysis
Source: PLoS One. 2015 Aug 28;10(8):e0135947. doi: 10.1371/journal.pone.0135947 (PMC4552710; doi:10.1371/journal.pone.0135947)
Supplement: S1 Table — (PDF) [file pone.0135947.s003.pdf]

S3 Table. Details of population, outcomes and study quality ratings.

Study ID	Population	Safety incident details	Multimorbidity details	Response rate at baseline 70%	Response rate at follow up 70%	Control for confounders
Bae et al. 2008 [35]	Civilian U.S. population	Receipt of recommended care for diabetes	Diabetes + A range of comorbid physical long term conditions	0	n/a	1
Barham et al. 2009 [36]	Patients eligible for lipid screening	Receipt of recommended Lipid screening	Dyslipidaemia +A range of comorbid physical long-term conditions	0	n/a	1
Beer et al. 2010 [37]	Community-dwelling older men	Markers of suboptimal and potentially inappropriate medication use	Multimorbidity score based on Charlson Index	0	n/r	0
Berger et al. 2009 [38]	Elderly patients with Generalized Anxiety Disorder	Potentially inappropriate prescribing of medication	Generalised anxiety disorder +	n/r	n/a	0
Bertomeu et al. 2009 [39]	Patients with Generalised Anxiety Disorder	Receipt of Optimal Medical Treatment (medication prescribing) for Coronary heart disease	Coronary Heart disease + Charlson Index	1	n/a	1
Blecker et al. 2010 [40]	Disabled Maryland Medicaid recipients	Receipt of recommended tests, care and prescription patterns	Heart failure + Severe Mental Illness	n/r	n/a	1
Bont et al. 2007 [41]	Elderly patients with exacerbations of chronic	GP adherence to guidelines suggesting prescribing antibiotics	Chronic Obstructive Pulmonary Disease + A range of comorbid long-	1	n/a	0

	pulmonary disease	in patients with exacerbations of COPD and comorbid conditions	term conditions including Diabetes type 1, Malignancies, Heart disease			
Calderon et al. 2012 [42]	Primary care patients	Adverse drug events such as allergic reactions , adverse effects and unintentional overdoses	Multimorbidity score based on the Adjusted Clinical Groups System	1	n/a	1
Calvert et al. 2009 [43]	Patients with heart failure	Receipt of recommended medication for the management of heart failure	Heart failure + Asthma or chronic obstructive pulmonary disease, diabetes, hypertension, heart disease	1	n/a	1
Chen et al. 2011 [44]	Patients presenting to the Emergency department	Adverse drug events such as allergic reactions , adverse effects and unintentional overdoses	Multimorbidity score based on Charlson Index	1	n/r	1
Classen et al. 2007 [45]	Home-based patients with self-reported stroke	Potential drug-drug interactions	Stroke + Count of comorbid physical long-term conditions	1	n/a	0
Dalton et al. 2011 [46]	Patients with diabetes registered in 23 general practices in London	Patterns of exclusions for three intermediate diabetes quality outcome indicators according to Quality and Outcomes Framework	Diabetes + A range of comorbid physical long-term conditions	1	n/a	1
Davis et al. 2008 [47]	Patient with heart failure with or without comorbid heart failure	Prescription of recommended cardiovascular drugs	Heart failure +Rheumatoid arthritis	0	n/a	0
Desai et al. 2005 [48]	Department of Veterans Affairs medical outpatients	Screening for alcohol disorders	Multimorbidity score based on Charlson Index	1	n/a	0
Desai et	Department of Veterans	Screening for depressive disorders	Multimorbidity score based on	1	n/a	0

al. 2006 [49]	Affairs medical outpatients	and follow-up evaluation	Charlson Index			
Druss et al. 2012 [50]	Medicaid enrolees with diabetes	Receipt of recommended diabetes tests and screening	Diabetes +Depression	n/r	n/a	1
Eguale et al. 2012 [51]	Primary care patients	Off-label prescribing	Multimorbidity score based on Charlson Index	n/r	n/a	1
Field et al. 2004 [52]	All Medicare enrolees cared for by a multispecialty group practice during 1 year	Adverse drug events including injuries resulting from the use of drugs	Charlson Index	n/r	n/a	0
Frigola et al. 2013 [53]	Primary care patients	Medication prescription errors	Chronic heart failure+ A range of comorbid physical long-term conditions	n/r	n/a	0
Goldberg et al. 2007 [54]	No-geriatric patients with a current diagnosis of type 2 diabetes	Quality of diabetes care	Diabetes + Severe Mental Illness such as schizophrenia and major mood disorder	n/r	n/a	1
Harman et al. 2004 [55]	Medical Expenditure Panel Survey respondents with depression	Receipt of depression care based on evidence-based treatment guidelines	International Classification of Diseases-9	n/r	n/a	1
Higashi et al. 2007 [56]	Patients participated three large community surveys	Adherence to quality indicators related to care processes	Multimorbidity score based on Charlson Index	n/r	n/a	0
Ho et al. 2006 [57]	Patients in a managed care organization (KPCO	Medication non-adherence	Diabetes + A range of comorbid physical long-term conditions	n/r	n/a	0

	diabetes registry)					
Kanner et al. 2012 [58]	Patients from five outpatient epilepsy clinics in the United States	Adverse drug events	Epilepsy+ Depression	1	n/a	0
Katerndahl et al. 2012 [59]	Patients with diabetes seen by the physician for at least 1 year	Medication adherence; Adherence to quality indicators for the management of diabetes	Diabetes + Depression	n/r	n/a	1
Ko et al. 2013 [60]	Patients in two rural communities-designated medically underserved areas	Poor glycaemic control	A composite score of the number of comorbidities (0-2 or 3-4)	n/r	n/a	0
Kontopantelis et al. 2013 [61]	Patients from 148 general practices	Quality of care was measured by achievement of the 17 diabetes quality indicators from the QOF	Diabetes + A range of physical long-term conditions	n/r	n/r	1
Krein et al. 2006 [62]	National data within the U.S. Department of Veterans Affairs health care system	Indicators of diabetes management based on recommended standards and Diabetes Quality Improvement Project	Diabetes +Depression	n/r	n/a	0
Lagomasino et al. 2005 [63]	Patients in 46 managed primary care practices screened positive for depression	Receipt and adherence to depression medication prescription guidelines:	Multimorbidity score based on Charlson Index	1	n/a	1

Lin et al. 2013 [64]	Medicare claims data for Alzheimer's disease and related disorders of Medicare beneficiaries	Hospital admissions preventable by good ambulatory care, or by early intervention to avoid severe disease	Dementia+ A range of physical long-term conditions	0	n/a	1
Lu et al. 2011 [65]	Commonwealth Fund International Health Policy Survey data among seven countries	Medication prescription errors	Multimorbidity score based on Charlson Index	n/r	n/a	1
Marcum et al. 2012 [66]	Older veterans with unplanned hospitalizations	Adverse drug reactions	Selim Physical comorbidity index	n/r	n/a	0
Mensah et al. 2007 [67]	A community-identified sample of people with epilepsy	Medication side effects	Epilepsy+ Anxiety	0	n/a	0
Mikuls et al. 2005 [68]	Primary care patients	Physician adherence to validated quality indicators for the treatment of gout and asymptomatic hyper-uraemia	Gout disease+ A range of physical long-term conditions including coronary artery disease, hypertension, diabetes, renal failure	n/r	n/a	1
Nasser et al. 2009 [69]	Patients with diabetes attending the diabetes clinics	Diabetes complications	Diabetes + Depression	n/r	n/a	0
Nuyen et al. 2005 [95]	A random sample of the practice population participated in an extensive health interview survey	Underdiagnosis of depression by general practitioners	International Classification of Primary Care	1	n/a	1

Obreli-Neto et al. 2012 [70]	Patients with at least one potential Drug-drug interaction	Adverse drug reactions	Multimorbidity score based on Charlson Index	0	1	1
Parchman et al. 2005 [71]	Veterans with one or more chronic illnesses	Medication side effects	Multimorbidity score based on count physical long-term conditions	0	n/a	1
Pawaskar et al. 2008 [72]	Patients with un-weighted health visits with insomnia	Optimal prescribing of medication	Insomnia + Charlson Index; Insomnia + Depression	n/r	n/a	1
Petersen et al. 2009 [73]	Veterans with hypertension who received primary care	Quality of care based on guidelines	Hypertension + A range of comorbid physical long-term conditions	0	n/r	1
Pugh et al. 2005 [74]	Veterans having at least one Veteran Affairs outpatient visit	Inappropriate prescribing	Multimorbidity score based on Charlson Index	n/r	n/a	1
Pugh, et al. 2010 [75]	Veterans with a new diagnosis of epilepsy	Potential drug–drug interactions	Epilepsy+ Depression; Epilepsy + a range physical long-term conditions	n/r	n/a	1
Reichard et al. 2012 [76]	Patients with physical disabilities and diabetes receiving medical benefits through Kansas Medicaid	Quality of Diabetes monitoring	Hypertension + Diabetes	n/r	n/a	1

Rigler 2004 et al. [77]	Community dwelling older adults	Inappropriate medication prescription based on Beers criteria	Cumulative Illness Rating Scale for Geriatrics	1	n/a	1
Ruigomez et al. 2007 [78]	Patients with chronic atrial fibrillation who were alive 1 month after initial diagnosis	Cerebrovascular accident	Oxford Medical Information System	1	1	1
Rupert, 2010 et al. [79]	Patients with diabetes who received care in primary care offices	Access (referral/non-referral) to Diabetes self-management Programme	Diabetes + Depression; Diabetes+ a range physical long-term conditions including coronary artery disease Hypertension, Hyperlipidaemia	0	n/a	0
Schnitzer et al. 2012 [80]	All complaints reporting on specific health care problems encountered in practice	Patients' complaints about the health care system and medical services are regarded as indicators of shortcomings in health care systems	Multimorbidity score based on count of physical long-term conditions	1	n/a	0
Shireman et al. 2010 [81]	Patients with developmental disabilities and diabetes who were enrolled in the Kansas Medicaid Aged, Blind and Disabled Program	Quality of care based on guidelines	Hypertension + Diabetes	n/r	n/a	1
Simeone et al. 2012 [82]	Patients with type 2 diabetes taking an oral antidiabetic agent	Hypoglycaemia-related emergency department visits	Diabetes + A range of comorbid physical long-term conditions	0	n/a	1
Simpson	Primary care patients	Exclusions from quality indicators	Stroke + A range of comorbid physical	n/r	n/a	1

et al. 2007 [83]	with stroke		long-term conditions			
Sloane et al. 2004 [84]	A stratified random sample of primary care facilities in Florida, Maryland, New Jersey, and North Carolina	Medication under-treatment and non-prescribing	Multimorbidity score based on count of physical long-term conditions	0	n/a	1
Thorpe et al. 2012 [85]	Medicare fee-for-service beneficiaries with a diagnosis of Diabetes Mellitus	Receipt of recommended monitoring tests for diabetes	Diabetes + Depression; Diabetes + A range of comorbid physical long-term conditions	n/r	n/a	1
Tomio et al. 2010 [86]	Beneficiaries with diabetes of National Health Insurance in two communities in Japan	Adherence to Diabetes quality indicators	Diabetes + Depression; Diabetes + A range of comorbid physical long-term conditions	1	n/a	1
Tsang et al. 2013 [87]	Patients at 457 English general practices obtained from the General Practice Research Database	Adverse events including injuries and poisoning	Multimorbidity score based on Expanded Diagnosis Clusters	1	n/a	1
van Dijk et al. 2007 [88]	Users of antidepressant, anti-hypertensive, or oral hypoglycaemic medication and their GPs	Medication adherence	Multimorbidity score based on Charlson Index	1	n/a	1
Weisman et al. 2007	Patients with rheumatoid arthritis and	Medically important infections	Rheumatoid arthritis+ Diabetes mellitus, chronic pulmonary disease	1	n/a	0

[89]	concomitant comorbidities					
Whooley et al. 2008 [90]	Outpatients with stable coronary heart disease	Medication adherence	Coronary Heart Disease + Depression	n/r	n/r	1
Wolff et al. 2002 [91]	Medicare beneficiaries	Potentially avoidable hospitalizations for ambulatory case sensitive condition	A composite score of the number of comorbidities (0-2 or 3-4)	1	n/a	1
Wong et al. 2011 [92]	Chinese patients attended a primary care clinic at least once, received an ICPC-2 code of T93 Lipid Disorders, and were prescribed a lipid-lowering agent in the territory	Medication adherence	Dyslipidaemia +A range of physical long-term conditions	n/r	n/a	1
Woodard et al. 2012 [93]	Patients with diabetes	Receipted diabetes care recommended by the American Diabetes Association	Diabetes + A range of physical long-term conditions	0	n/r	1
Zwar et al. 2011 [94]	Patients and general practitioners from 44 practices participated in the study	Misdiagnosis of Chronic Obstructive Pulmonary Disease	Chronic Obstructive Pulmonary Disease + A range of physical long-term conditions	0	n/a	1

Note: n/a= not applicable; n/r= not/reported
